# Descriptive Anatomy and Three-Dimensional Reconstruction of the Skull of the Early Tetrapod *Acanthostega gunnari* Jarvik, 1952

**DOI:** 10.1371/journal.pone.0118882

**Published:** 2015-03-11

**Authors:** Laura B. Porro, Emily J. Rayfield, Jennifer A. Clack

**Affiliations:** 1 Structure and Motion Laboratory, Royal Veterinary College, University of London, Hatfield, United Kingdom; 2 School of Earth Sciences, University of Bristol, Bristol, United Kingdom; 3 Department of Zoology, University of Cambridge, Cambridge, United Kingdom; College of the Holy Cross, UNITED STATES

## Abstract

The early tetrapod *Acanthostega gunnari* is an iconic fossil taxon exhibiting skeletal morphology reflecting the transition of vertebrates from water onto land. Computed tomography data of two *Acanthostega* skulls was segmented using visualization software to digitally separate bone from matrix and individual bones of the skull from each other. A revised description of cranial and lower jaw anatomy in this taxon based on CT data includes new details of sutural morphology, the previously undescribed quadrate and articular bones, and the mandibular symphysis. Sutural morphology is used to infer loading regime in the skull during feeding, and suggests *Acanthostega* used its anterior jaws to initially seize prey while smaller posterior teeth were used to restrain struggling prey during ingestion. Novel methods were used to repair and retrodeform the skull, resulting in a three-dimensional digital reconstruction that features a longer postorbital region and more strongly hooked anterior lower jaw than previous attempts while supporting the presence of a midline gap between the nasals and median rostrals.

## Introduction

The invasion of the land by vertebrates was a key moment in evolutionary history, leading to worldwide colonization of terrestrial environments by four-limbed tetrapods. Areas of central East Greenland, composed of Middle and Upper Devonian rocks, have yielded several iconic Late Devonian (Famennian) tetrapods. *Ichthyostega* was the first named genus from East Greenland [1] followed by *Acanthostega* [2] and *Ymeria* [3]. One of the oldest known tetrapods, *Acanthostega gunnari* is represented by a number of specimens and is crucial for understanding the anatomy, ecology and evolution of early tetrapods, having been incorporated into studies on feeding [4], [5], [6], [7], and locomotion [8], [9].

Several anatomical descriptions of the *Acanthostega* skull are available [2], [10], [11], including studies focused on the skull roof [12], palate [13], braincase [13], [14], [15], [16], and lower jaw [17]. These descriptions have served as the basis for reconstructions of the *Acanthostega* cranium in lateral, dorsal and ventral views [2], [12], and of the lower jaw [17]. New information from the Late Devonian genus *Ventastega* from Latvia [18] resulted in a revised reconstruction of the *Acanthostega* cranium featuring a midline gap between the frontal and nasal bones, and between the nasals and median rostrals [11], [19]. However, no single specimen of *Acanthostega* preserves a skull that is complete, articulated and undistorted, limiting attempts to reconstruct the jaw adductor musculature (as in the baphetid *Megalocephalus* [20], [21]) or carry out quantitative, three-dimensional (3D) biomechanical analyses.

Computed tomography (CT) is increasingly being applied to fossils to prepare fragile or small material digitally [22], [23], visualize internal cavities and bone histology [24], [25], and capture skeletal morphology for biomechanical analyses [26], [27]. In this study, we used CT scanning and visualization software to prepare specimens of *Acanthostega* digitally and produce a new osteological description of the *Acanthostega* skull, supplementing and amending previous descriptions [2], [10], [11], [12], [13], [14], [15], [16], [17]. Individual bones from CT data were then digitally manipulated to produce the first 3D computer model of the *Acanthostega* skull.

## Materials and Methods

**Fig 1:**
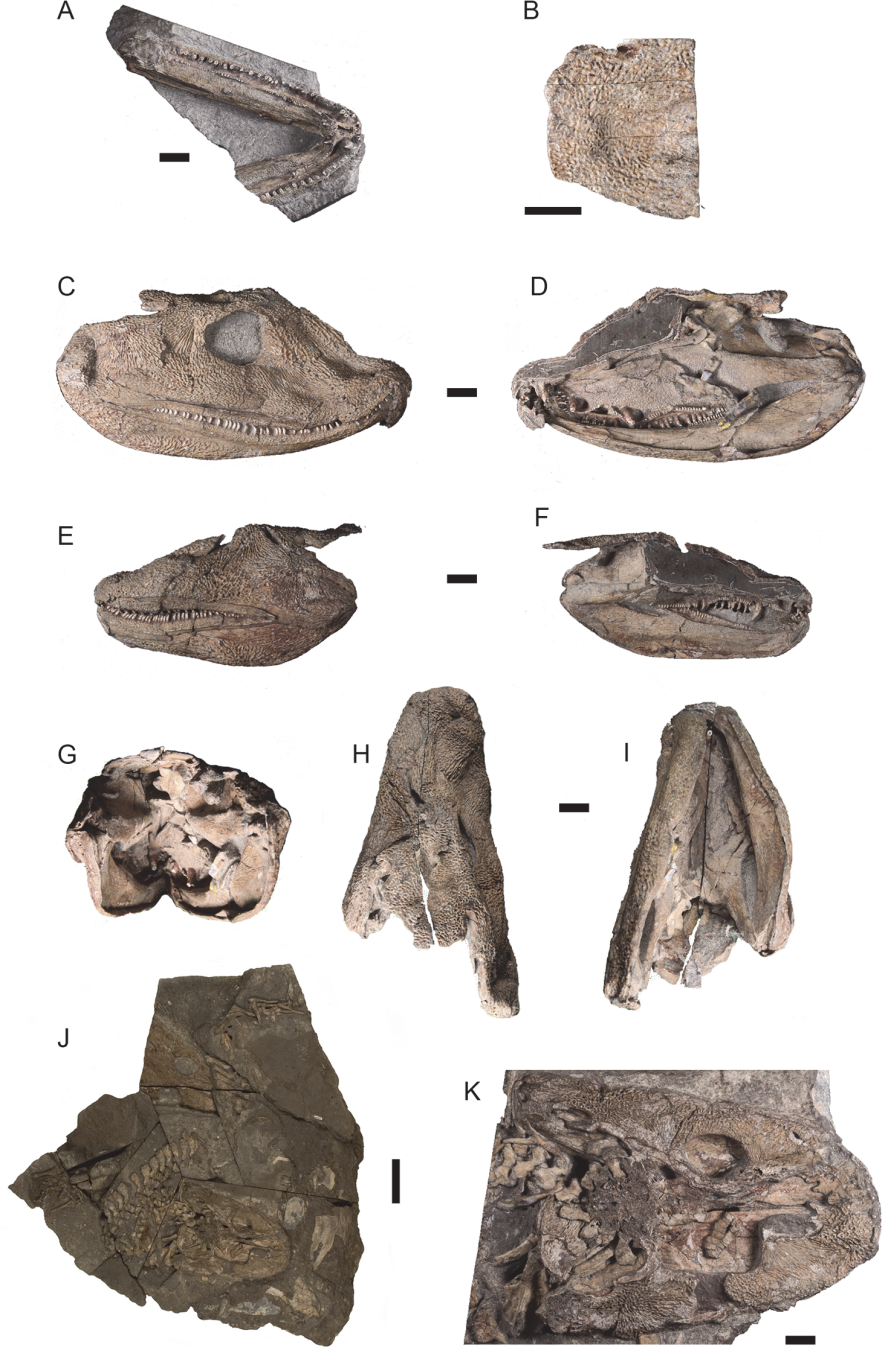
Photographs of original *Acanthostega gunnari* specimens. Dorsal views of the incomplete, articulated lower jaws (A) and posterior skull table (B) of MGUH-VP-8158 (“Rosie”). Lateral (C) and medial (D) views of the right side of MGUH-VP-8160 (“Grace”); lateral (E) and medial (F) views of the left side of MGUH-VP-8160; and posterior (G), dorsal (H) and ventral (I) views of both halves of MGUH-VP-8160. Dorsal view (J) of the large slab, containing MGUH 29019 (“Boris”, bottom center) and MGUH-VP-8161 (“Uncle Fred”, upper left). Close-up MGUH 29019 in dorsal view (K). All scale bars equal 10 mm except for the scale bar in K, which equals 50 mm.

Three *Acanthostega gunnari* skulls were CT-scanned for this study. All three specimens pertain to the Geological Museum of the University of Copenhagen, København, Denmark (MGUH) and are currently on long-term loan to the University Museum of Zoology Cambridge, Cambridge, United Kingdom (UMZC, also known as CAMZM). These specimens were collected under the 1987 auspices of the Greenland Geological Survey in Denmark; field permits were obtained by the late Dr. Svend Erik Bendix-Almgreen of the MGUH. MGUH-VP-8158 (‘Rosie’, formerly referred to by the field number MGUH f.n. 1300a), an incomplete skull that includes incomplete, articulated lower jaws (Fig. 1A) and the skull table overlying a well-preserved braincase (Fig. 1B); MGUH-VP-8160 (‘Grace’, formerly referred to by the field number MGUH f.n. 1300b), consisting of a nearly complete, mediolaterally compressed skull in which the anterior snout has been sheared to the right (Fig. 1C–1I); and MGUH 29019 (‘Boris’, formerly referred to by the field number MGUH f.n. 1227), a dorsoventrally flattened skull lacking most of the skull table (Fig. 1J and 1K). Prior to scanning (between 1989 and 1995), both specimens were mechanically prepared using a dental mallet, pneumatic pen (compressed air-driven reciprocating hammer action) and a 1 mm hand-held tungsten carbide needle held in a pin vice, for which the tip was regularly sharpened on a mechanical grind stone. All work was carried out under a stereomicroscope. Various portions of the upper jaw of MGUH-VP-8158 were separated from the lower jaws. The skull table of MGUH-VP-8158 was sectioned anteroposteriorly using a Well Diamond Wire Saw (Norcross, Georgia, USA) with a 0.3 mm wire; the wire passed through the left parietal, supratemporal, tabular and postparietal, and the two portions were reassembled prior to CT-scanning by JAC using Paraloid B-72. Additionally, the symphysis of MGUH-VP-8158 was sectioned, the wire passing between the left and right dentaries and through the left splenial and right adsymphysial. MGUH-VP-8160 was sectioned into right and left halves along the sagittal midline in order to access the interior of the skull (S. Finney, personal communication). The skull of MGUH 29019, which is in articulation with the nearly complete post-cranial skeleton of this individual, was separated from the main block. The block also contains the isolated skull of a second individual (MGUH-VP-8161, ‘Uncle Fred’, Fig. 1J).

**Fig 2:**
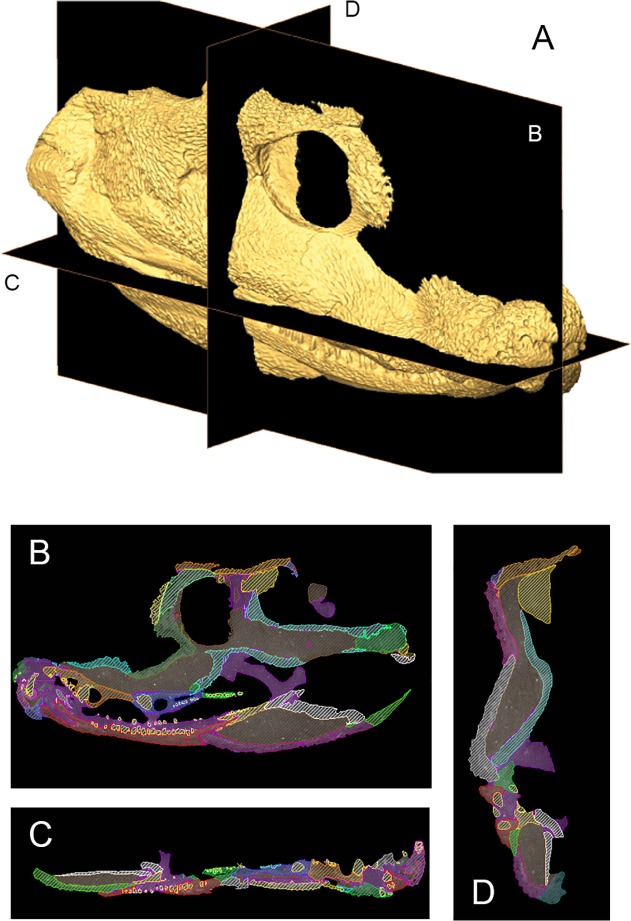
Segmentation of CT data of *Acanthostega gunnari*. Isosurface (A) of (MGUH-VP-8160) with labeled sagittal (A), frontal (B) and transverse (C) planes. These slices are shown in insets, with shaded areas representing individual bones segmented from each other and the surrounding matrix.

**Fig 3:**
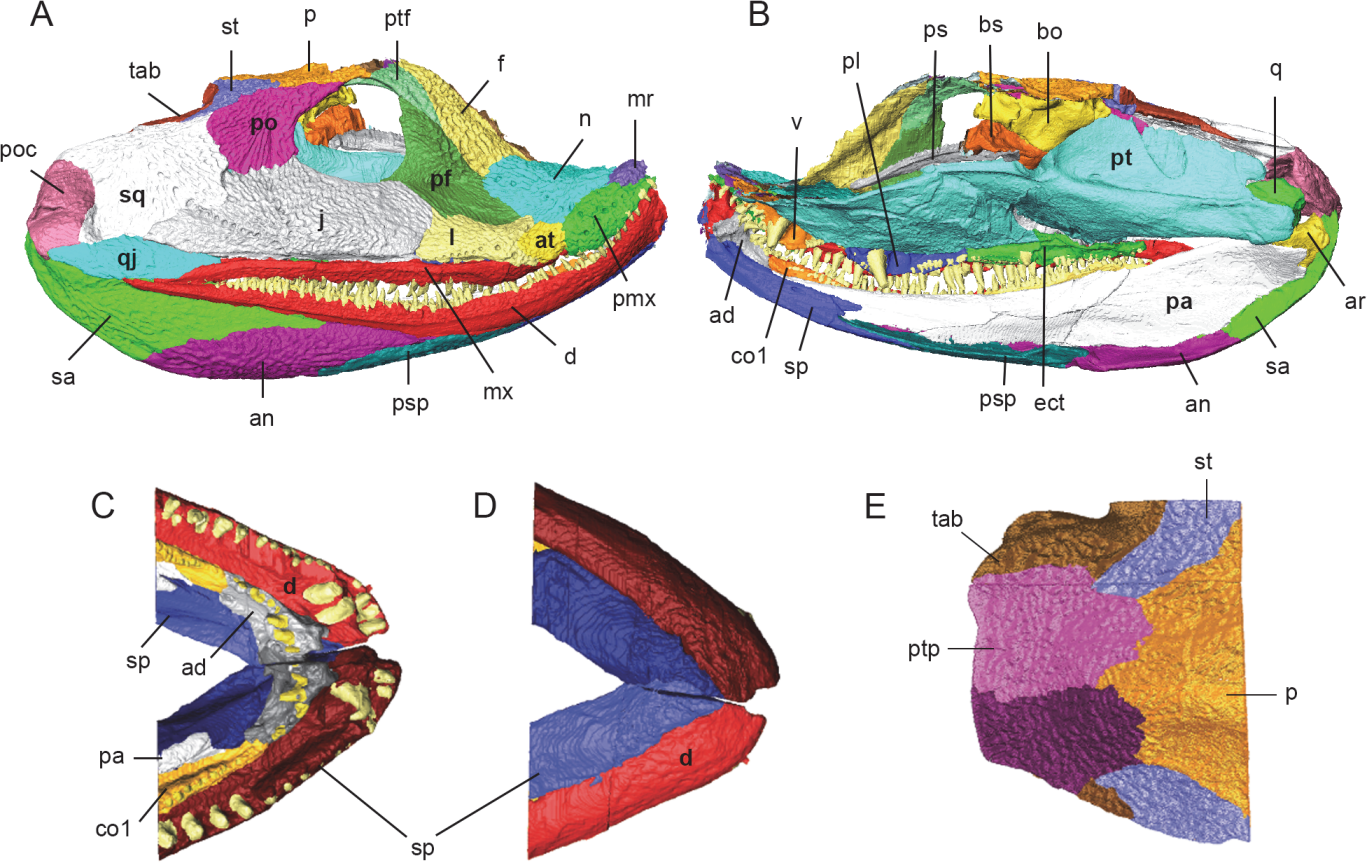
Surface models of *Acanthostega gunnari* prior to retrodeformation. Lateral (A) and medial (B) views of the right side of MGUH-VP-8160. Dorsal (C) and ventral (D) views of the anterior ends of the lower jaws of MGUH-VP-8158. Dorsal (E) view of the skull roof of MGUH-VP-8158. Individual bones are shown in various colours. Anatomical abbreviations: ad, adsymphysial; an, angular; ar, articular; at, anterior tectal; bo, basioccipital; bs, basisphenoid; co1, coronoid 1; d, dentary; ect, ectopterygoid; f, frontal; j, jugal; l, lacrimal; mr, median rostral; mx, maxilla; n, nasal; p, parietal; pa, prearticular; pf, prefrontal; pl, palatine; pmx, premaxilla; po, postorbital; poc, preopercular; ps, parasphenoid; psp, postsplenial; pt, pterygoid; ptf, postfrontal; ptp, postparietal; q, quadrate; qj, quadratojugal; sa, surangular; sp, splenial; sq, squamosal; st, supratemporal; tab, tabular; v, vomer.

The lower jaws of MGUH-VP-8158 and the right half of MGUH-VP-8160 were scanned in 2007 at Nikon Metrology (formerly X-Tek Systems Ltd. [Metris]) in Tring, United Kingdom on an X-Tek H 225 ST CT-scanner. The skull table of MGUH-VP-8158, left half of MGUH-VP-8160, and skull of MGUH 29019 were scanned in 2014 in the Cambridge Biotomography Centre (Zoology Department) at the University of Cambridge on an X-Tek H225 CT scanner. Reconstruction of the lower jaws of MGUH-VP-8158 produced 1590 slices (oriented obliquely through the specimen) composed of isometric voxels with a resolution of 0.0517 mm/voxel; reconstruction of the skull table of MGUH-VP-8158 produced 1616 transverse slices composed of isometric voxels with a resolution of 0.035 mm/pixel; reconstruction of the right side of MGUH-VP-8160 produced 1489 transverse slices composed of isometric voxels with a resolution of 0.0964 mm/voxel; reconstruction of the left side of MGUH-VP-8160 produced 1893 transverse slices composed of isometric voxels with a resolution of 0.053 mm/pixel; reconstruction of the skull associated with the postcranial skeleton of MGUH 29019 produced 1915 transverse slices composed of isometric voxels with a resolution of 0.081 mm/pixel. CT scans were processed using the 3D visualization software package Avizo 7.1.1 (FEI Visualization Sciences Group, Mérignac Cédex, France) on a 64-bit Dell Inspiron 15R laptop with 8 GB RAM. Within the segmentation editor, density thresholding was initially used to separate bone from matrix (Fig. 2). Scans were then processed slice-by-slice (interpolating across no more than five slices at a time) to separate individual bones, teeth and sutures (Fig. 3). Sutures typically present as low density areas between bones, although occasionally high density minerals precipitated within sutures. Original specimens (currently housed at the University Museum of Zoology Cambridge) were used to confirm the location of sutures and differentiate them from post-mortem damage. Individual bones were isolated and separately labeled within the segmentation editor and 3D surface models (.surf files) of each element were created that could be manipulated in isolation in 3D space; the following anatomical description is based on these models.

Some limits to the data sets used in the description and 3D reconstruction should be noted. Only CT scans of MGUH-VP-8158 and the right side of MGUH-VP-8160 were fully segmented. This was due to strong distortion of the left side of MGUH-VP-8160 and dorsoventral crushing and loss of the skull table in MGUH 29019; nonetheless, both of these specimens (as well as additional MGUH specimens, specified below) provided descriptive information. The left lower jaw ramus of MGUH-VP-8158 is broken at the level of the anterior limit of the mandibular adductor fossa and the right lower jaw ramus is broken at the posterior end of the anterior coronoid; this specimen was used primarily for understanding the morphology of the mandibular symphysis. The skull table of MGUH-VP-8158 is preserved to a point posterior to the parietal foramen and both lateral margins are missing. Only the parasphenoid, basisphenoid, basioccipital and left stapes are well-preserved in MGUH-VP-8160; other elements of the braincase are absent or fragmentary.

Computer reconstructions of fossil skulls are typically based on complete, articulated and minimally deformed specimens. A few studies have digitally reconstructed fossil skulls from disarticulated specimens [28], [29], [30] but, in most cases, precise methodological details are not reported. Reconstructions of fossil hominid skulls frequently use the skull of *Homo sapiens* as a ‘template’ upon which fragments are assembled [31]; however, as no early tetrapod taxon closely related to *Acanthostega* features a complete, undistorted skull, the use of such a ‘template’ is not possible.

Nearly all of the bones used in the 3D reconstruction are from the right side of MGUH-VP-8160, with the exception of the postparietals (see below). As MGUH-VP-8160 is mediolaterally compressed, the intramandibular angle (48°) of MGUH-VP-8158 (which we assume is minimally distorted) was measured on the original specimen and used as a guide for initial reconstruction. The right lower jaw of MGUH-VP-8160 was digitally duplicated and reflected across the sagittal midline in Avizo and the lower jaws set at an intramandibular angle of 48°. Using the Transform Editor within Avizo, the teeth of the upper jaws (and their associated bones) were placed in their correct positions relative to the teeth of the lower jaws—as preserved in fossils, the premaxillary/maxillary tooth row is located external (= buccal) to the marginal dentary tooth row, and the palatal tooth row fits between the dentary and adsymphysial/coronoid tooth rows. The cranium was then built upwards by fitting the remaining cranial elements together at sutural contacts. The complete left postparietal of MGUH-VP-8158—along with portions of the surrounding supratemporal, tabular and parietal—was reflected to become a right side element, scaled by a factor of 0.32 (obtained from elements shared between the two CT data sets, such as supratemporal and parasphenoid), and incorporated into the 3D reconstruction. Right side elements were duplicated and reflected to form the left half of the cranium.

**Fig 4:**
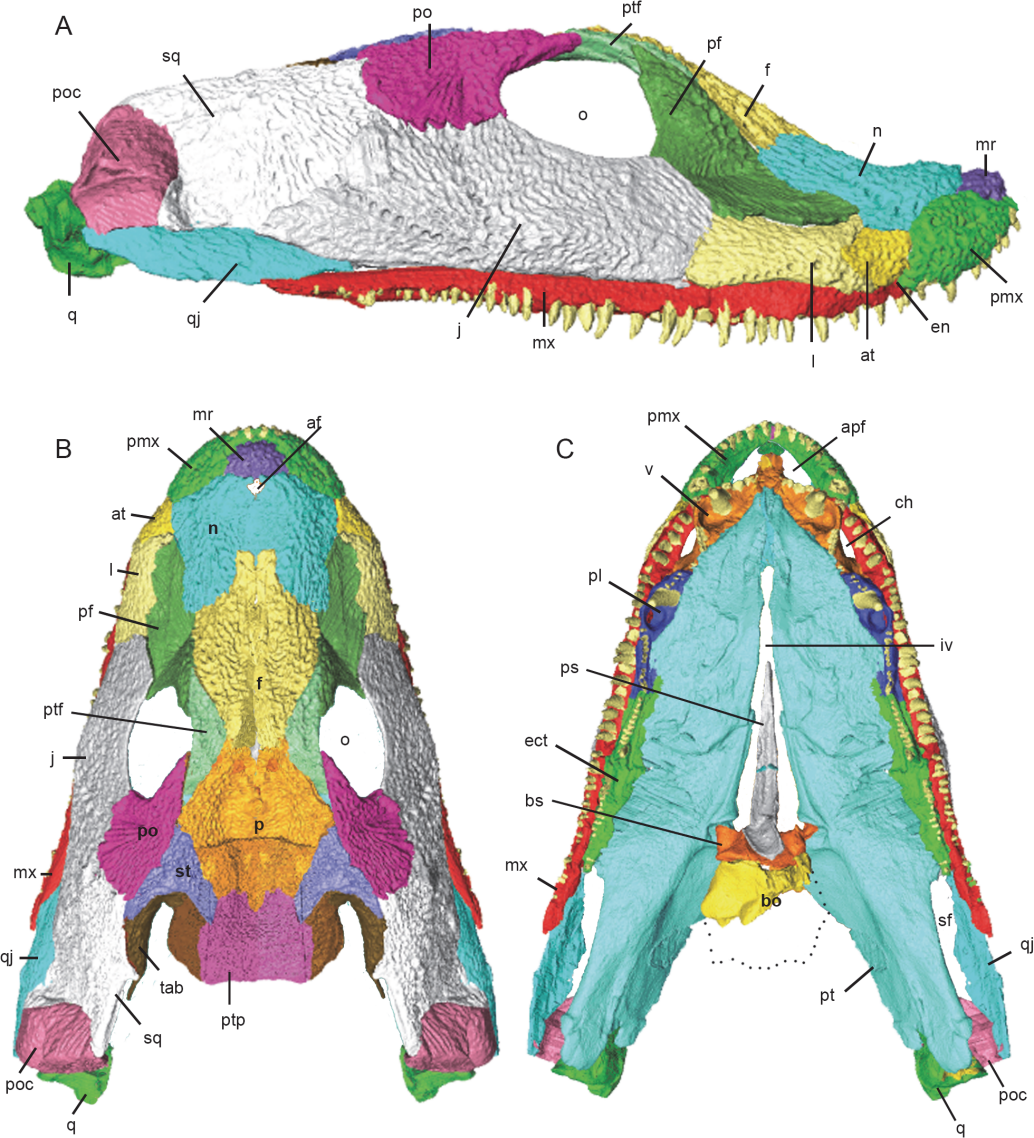
3D reconstruction of the cranium of *Acanthostega gunnari*. Lateral (A), dorsal (B) and ventral (C) views of the cranium. Individual bones are shown in various colours. Anatomical abbreviations: af, anterior fontanelle; apf, anterior palatal fossa; at, anterior tectal; bo, basioccipital; bs, basisphenoid; ch, choana; ect, ectopterygoid; en, external naris; f, frontal; iv, interpterygoid vacuity; j, jugal; l, lacrimal; mr, median rostral; mx, maxilla; n, nasal; o, orbit; p, parietal; pf, prefrontal; pl, palatine; pmx, premaxilla; po, postorbital; poc, preopercular; ps, parasphenoid; pt, pterygoid; ptf, postfrontal; ptp, postparietal; q, quadrate; qj, quadratojugal; sf, subtemporal fossa; sq, squamosal; st, supratemporal; tab, tabular; v, vomer. Dotted outline in C shows approximate outline of basioccipital.

**Fig 5:**
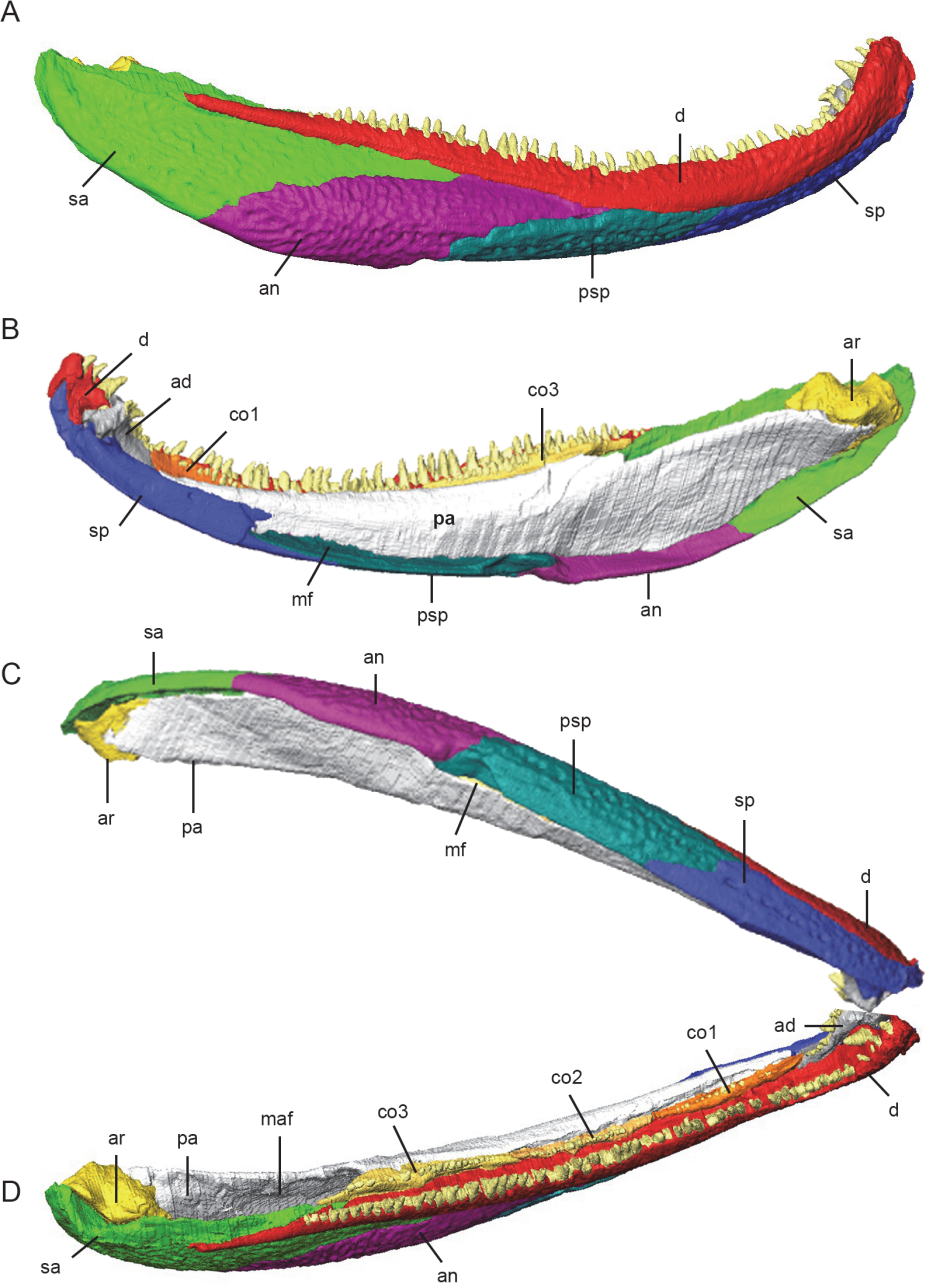
3D reconstruction of the lower jaw of *Acanthostega gunnari*. Lateral (A), medial (B), ventral (C) and dorsal (D) views of the right lower jaw. Individual bones are shown in various colours. Anatomical abbreviations: ad, adsymphysial; an, angular; ar, articular; co1, coronoid 1; co2, coronoid 2; co3, coronoid 3; d, dentary; maf, mandibular adductor fossa; mf, Meckelian fenestra; pa, prearticular; psp, postsplenial; sa, surangular; sp, splenial.

The pterygoid bones were reserved as a final test of the reconstruction. Fossil specimens demonstrate that the pterygoids of *Acanthostega* meet closely at the midline anteriorly, separating posteriorly to allow the parasphenoid to fit between them. The pterygoids must also articulate correctly with the marginal palatal bones (vomer, palatine, ectopterygoid) and the basipterygoid processes of the braincase. When the pterygoids were initially fitted into the reconstruction, they overlapped slightly in the midline, suggesting that the reconstructed skull was too narrow. The intramandibular angle was gradually increased to 53.4°, at which point the pterygoids articulated properly with each other and surrounding elements, resulting in a slightly wider and flatter skull (Figs. 4 and 5). Transformation matrices for all skull bones from the original data set to the final, 3D reconstruction are available as S1 Matrix; a 3D PDF of the reconstructed skull is available for personal inspection as S1 Model. It should be noted that the 3D model represents our hypothesis of the reconstructed skull of *Acanthostega*, based on available specimens, scan resolution and personal interpretation.

There is little breakage or deformation of individual bones in MGUH-VP-8160, with two exceptions. The right prearticular is broken into three pieces: a long anterior segment articulated to surrounding bones (dentary, splenial, adsymphysial and coronoid), and middle and posterior fragments pushed laterally into the Meckelian canal and mandibular adductor fossa. These surfaces were manipulated in 3D using the Transform Editor and their edges fitted against the undeformed anterior segment. Breaks were manually repaired in the Segmentation Editor using interpolation. Additionally, the right pterygoid features a tab of bone at its lateral margin (level with the midpoint of the ectopterygoid) that has been partially broken away and deformed relative to the main body of the pterygoid. This breakage and deformation is localized and does not affect the pterygoid’s articulations with adjacent bones.

## Results

### Facial skeleton

The preserved portion of the left lower jaw ramus of MGUH-VP-8158 measures 88 mm total length; the skull table of MGUH-VP-8158 has a maximum anteroposterior length of 45 mm; the skull of MGUH-VP-8160 measures 111 mm from the anterior tip of the right median rostral to the posterior tip of right preopercular. As preserved, the orbit of MGUH-VP-8160 measures 16 mm tall (at its midpoint) and 18 mm in length. (For further comments on skull morphology, see section titled “3D reconstruction of the *Acanthostega* skull” below.) Unless noted, all bones are right side elements from MGUH-VP-8160.

The presence of one or paired **median rostrals** (= internasals) was predicted by the shape of the nasals [2] and premaxilla [10], with the element eventually described by Clack [13]. Both left and right median rostrals are preserved in MGUH-VP-8160 and fit into an embayment over the narrow, anterior process of the premaxilla (Figs. 3A and 4). Scans demonstrate that the ventral margin of the median rostral overlaps the dorsal edge of the premaxilla in a short scarf joint and that the right and left median rostrals contact each other at the midline in a vertical butt joint. The posterolateral edge of the median rostral makes a weak contact with the anterior margin of the nasal.

The **premaxilla** of *Acanthostega* was first described by Clack [10], [13]. The body of the premaxilla is oval-shaped in lateral view with a dorsoventrally narrow anterior process (Figs. 3A and 4). In transverse section, the body of the premaxilla is widest at the tooth bases (where it projects medially as a short shelf) and tapers dorsally. Both the external and internal surfaces of the premaxilla are laterally convex. CT scans reveal ten teeth preserved in the right and left premaxillae of MGUH-VP-8160, although gaps suggest more teeth were originally present [10], [12], [13]. The premaxillary teeth generally increase in size posteriorly and are medially curved.

CT scans demonstrate that the dorsal margin of the anterior process of the premaxilla is overlapped by the median rostral; posteriorly, the premaxilla overlaps the ventral edge of the nasal in a short scarf suture. Due postmortem deformation, the midline joint between the anterior processes of the premaxillae has opened, but it appears this was a simple butt joint. We found that the anteromedial process of the left vomer dorsally laps the internal shelf of the premaxilla in an interdigitating suture; posterior to the anterior palatal fossa, the rounded lateral shelf of the vomer inserts above the medial shelf of the premaxilla. CT scans show that the anterior tip of the maxilla wedges between the premaxilla (lateral) and vomer (medial), contacting both bones and excluding the premaxilla from the choana (Fig. 4C). The rounded posterior border of the premaxilla overlaps the anterior margin of the anterior tectal. Internal to this contact, the posterior surface of the premaxilla is concave and smooth, forming the anterior wall of the naris. The supraorbital and orbital lateral line canals meet in a Y-shape on the lateral surface of the premaxilla and continue onto the median rostral as described by Clack [13].

The **anterior tectal** (Figs. 3A and 4) of *Acanthostega* was first described by Clack [13]. Posteroventrally, it overlaps the dorsal edge of the maxilla in a short scarf; dorsally, it contacts the nasal at a rounded butt joint. The rounded posterior margin of the anterior tectal fits into an embayment in the anterior edge of the lacrimal, overlapping the lacrimal along this entire contact in a short scarf. As a result of these overlapping contacts, the anterior tectal is a superficial bone that is smaller in internal view than in external view. The anteroventral margin of the anterior tectal is free between its contacts with the premaxilla and maxilla, forming the lateral wall of the naris and excluding the nasal and lacrimal.

The **maxilla** (Figs. 3A and 4) is a long, narrow bone preserving 44 teeth, although a number of gaps suggest more teeth were originally present [13]. The first two maxillary teeth (in the anterior process, see below) lie medial to the posterior premaxillary teeth. The largest maxillary teeth are at positions 7–25. All of the maxillary teeth are rounded in cross section, posteriorly recurved (in lateral view) and medially curved.

In transverse section, the external surface of the body of the maxilla dorsal to the tooth row is strongly laterally convex. Anteriorly, the maxilla tapers and curves dorsally and medially [2], [13] to form a mediolaterally thin anterior process that bears small teeth and inserts between the premaxilla and vomer. Posterior to these contacts, the anterior process of the maxilla separates the naris and choana, forming the internal wall of the former and the external wall of the latter (Fig. 4C). We found that the dorsal margin of the maxilla is overlapped by the anterior tectal; below this contact is a prominent sensory pore [13]. Posterior to this pore, the maxilla widens dorsoventrally and mediolaterally; this portion of the maxilla bears the largest teeth. The dorsal margin of the maxilla forms long butt joints with the ventral edges of the lacrimal and jugal. Posteriorly the maxilla tapers to a point ventral to the quadratojugal, which it contacts at a rounded butt joint. The butt joints between the maxilla and the lacrimal, jugal and quadratojugal, and the fact the maxilla has separated from these bones in the type skull [2], suggests a loose contact between these elements.

Posterior to the choana, the medial surface of the maxilla dorsal to the tooth row projects inwards, forming a rounded shelf (= palatal lamina of Jarvik [2]; = palatal process of Clack [13]). Anteriorly, this shelf is overlapped by the posterior tip of the vomer. Posteriorly, the lateral process of the palatine overlaps the medial shelf of the maxilla; some fine interdigitations are visible at this contact in CT scans. In the center of maxilla-palatine junction, there is also broad contact between the lateral surface of the palatine (the lateral ridge that borders the replacement pit) and the medial surface of the maxilla. The contact between the medial surface of the maxilla and lateral surface of the ectopterygoid is a simple butt joint.

The **lacrimal** (Figs. 3A and 4) is roughly rectangular in lateral view; in transverse section it is mediolaterally thin and laterally bowed. The anterior margin is embayed to accommodate the anterior tectal, forming short, rounded dorsal and ventral prongs that underlap the anterior tectal. The dorsal prong overlaps the ventral margin of the nasal in a short scarf joint. A similar spike-like projection of the lacrimal onto the nasal was described in MGUH f.n. 1274 [12]. The lacrimal features an arched row of ten pores (part of the infraorbital lateral line canal), the anterior pores being larger than posterior pores [12]. The posterior margin of the lacrimal contacts the jugal in an interdigitating suture; in external view, the contact is weakly anteriorly convex. The dorsal margin of the lacrimal contacts the prefrontal in a simple butt joint; thus, the jugal and prefrontal exclude the lacrimal from the orbital margin in MGUH-VP-8160, although this is not the case in other *Acanthostega* specimens [12].

The **jugal** (Figs. 3A and 4) is a major bone of the cheek region and forms the ventral margin of the orbit. The jugal is longer than it is deep; in transverse section it is strongly bowed laterally [12] with a thickened ventral margin. Posteriorly, we found that the jugal tapers to a point that overlaps the quadratojugal (ventrally) and the squamosal (dorsally). The maxilla and quadratojugal exclude the jugal from the ventral margin of the cranium. Although the posterior tip of the jugal overlaps the squamosal, the posterodorsal margin of the jugal is extensively overlapped by an anteroventrally projecting tongue of the squamosal. The dorsal margin of the jugal underlaps the ventral margin of the postorbital. Both the jugal-squamosal and jugal-postorbital sutures are interdigitated in external view. The anterodorsal margin of the jugal overlaps the prefrontal in a short scarf joint. Therefore, the jugal does not underlap all surrounding bones in MGUH-VP-8160 as reported by Clack [12]. The infraorbital lateral line canal continues onto the jugal from the lacrimal; there are six or seven small pores on the ventral margin of the jugal immediately dorsal to its contact with the maxilla. The line then splits: the upper branch, containing approximately six pores, rises towards the postorbital; lower branch, containing ten pores, continues onto the squamosal.

The **squamosal** (Figs. 3A and 4) was described in detail by Clack [10], [12] and is a large bone contributing to the cheek and posterior margin of the skull. The anterodorsal margin of the squamosal meets the ventrolateral margins of the supratemporal and tabular bones in a unique, curving lap surface [12]. CT scans also reveal a medial lamina of the squamosal that extensively underlaps the ventral surfaces of the supratemporal and tabular near its anterodorsal tip. Midway along the dorsal margin of the squamosal there is a medially projecting prong (Fig. 4B) that externally overlaps the tabular [12]. Posterior to this prong the squamosal and tabular bones separate, producing an embayment. The medial margin of the squamosal thickens and turns inwards, creating a flange that forms the free posterior edge of the cranium. The squamosal meets the preopercular in an anteriorly convex, interdigitated suture along its posterior margin. We found that the ventral margin of the squamosal contacts the dorsal margin of the quadratojugal in a rounded butt joint. The anterodorsal margin of the squamosal is embayed to receive the postorbital; dorsally, the postorbital externally overlaps the squamosal while ventrally the squamosal overlaps the postorbital. The squamosal-postorbital suture is interdigitated along its entire length.

The lateral line of the jugal continues onto the lateral surface of the squamosal as six large pores. This line then turns abruptly and a large number of smaller pores occur along the free dorsal margin of the squamosal.

The **quadratojugal** is dorsoventrally tallest at its midsection, tapering anteriorly and posteriorly (Figs. 3A and 4). In transverse section it is weakly laterally convex. Its anterior process is overlapped by the jugal—it does not appear to overlap the jugal as described by Clack [12]. Posteriorly, ventral edge of the preopercular overlaps the dorsal margin of the quadratojugal. There appears to be no contact between the quadratojugal and the quadrate.

The **preopercular** is an oval-shaped bone forming the posteroventral corner of the cranium (Figs. 3A, 3B and 4). In MGUH-VP-8160, the preopercular has two distinct external faces: a larger surface directed laterally and a smaller surface directed posterodorsally. The dorsal margin of the preopercular is thickened and inturned, continuous with a similar flange on the squamosal and forming the free edge of the rear of the cranium. CT scans reveal a deep notch filled with matrix within the body of the preopercular—this cavity is likely damage due to deformation of the skull and marks where a dorsolateral sheet of bone has split from the main body of the preopercular. The internal surface of the preopercular broadly contacts the lateral surface of the quadrate. The lateral line pores of the squamosal continue onto the preopercular, with four large pores opening on the posterodorsal face.

The **quadrate** (Fig. 4) of *Acanthostega* has been only superficially described [10], [13] as no specimen clearly shows this bone. CT-scans demonstrate that the right quadrate is roughly triangular in shape in lateral view, being tallest posteriorly and tapering anteriorly. In ventral view, the anterior quadrate is mediolaterally narrow but expands dramatically posteriorly to form the jaw joint. In posterior view, it is triangular with a wide base. The narrow, dorsal edge of the quadrate appears to be free from contacts along most of its length. The dorsolateral face of the quadrate loosely contacts the medial face of the preopercular. There is no contact between the quadrate and the quadratojugal or squamosal. The dorsomedial face of the quadrate is overlapped extensively by the quadrate ramus of the pterygoid. The articulation surface features two condyles (lateral and medial) divided by a groove that is transversely narrow and deep anteriorly and gradually widens and becomes shallow posteriorly. A small, rounded pit on the posteoventral surface of the quadrate corresponds to the ligament pit described by Clack [10].

### Skull roof

The **nasals** (Figs. 3A and 4) are roughly rectangular in dorsal view and dorsally arched in transverse section. The nasals contact each other at the midline in a weak, short vertical butt joint posteriorly; anteriorly, there is no midline contact between the nasals. The posterior margin of the nasal extensively overlaps the anterior margin of the prefrontal, contrary to the relationship reported by Clack [12]. The contact between dorsal edge of the nasal and the anterior margin of the frontal is strongly interdigitated in external view. The right nasal overlaps the right frontal; however, this is likely due to deformation that has pushed the frontal under the nasal. In contrast, the left frontal slightly overlaps the left nasal in an externally interdigitated contact. The supraorbital lateral line traverses the lateral margin of the nasal as eight or nine large pores before continuing onto the frontal.

The **prefrontal** is a triangular bone forming the anterior margin of the orbit (Figs. 3A and 4). In transverse section the prefrontal is mediolaterally wide dorsally and narrows ventrally. The right prefrontal of MGUH-VP-8160 features a depression or groove oriented obliquely across the bone that divides the external surface into a steeply oriented face anterior to the orbit and a more horizontal portion along the side of the snout. (The thickened external ridge described by Clack [12] lies dorsal and medial to this groove.) We found that this feature is represented on the internal surface of the prefrontal as a sharp ridge originating at the anteroventral corner of the orbit and directed anteriorly. Deformation has pushed the left lacrimal of MGUH-VP-8160 upwards, obscuring the ventral portion of the prefrontal. However, CT scans reveal it is identical to the right prefrontal in cross-sectional geometry and also features a distinct ‘kink’, representing the external groove (and its corresponding internal ridge). The groove dividing the prefrontal into two faces is also clearly visible on the left side of MGUH 29019 (Fig. 1K, the right prefrontal of this specimen is incomplete) and appears to be present on the partially exposed left prefrontal of MGUH-VP-8161 (Fig. 1J). CT scans of MGUH-VP-8160 and MGUH 29019 clearly demonstrate that this ‘kink’ is genuine and not due to breakage. The prefrontal tapers anteriorly and ventrally as a process that passes between the nasal and lacrimal, approaching but not contacting the dorsal margin of the anterior tectal. The anterolateral margin of the frontal overlaps the prefrontal while the postfrontal overlaps the posterodorsal margin of the prefrontal in an extensive, interdigitated suture. As described by Clack [12] the orbital margin of the prefrontal bears a small lip, which projects into the orbit (Fig. 3A).

The **frontal** is an elongate, parallel-sided bone with a tapering posterior tip (Figs. 3A and 4). The left frontal overlaps the right frontal along the entire midline contact of the two elements, the degree of overlap increasing anteriorly. This is due to deformation of the skull and the original nature of the frontal-frontal contact is difficult to discern in this specimen. Based on the exposed medial margin of the frontal, it appears this contact was either a butt joint or a short overlap. The medial margin of the postfrontal is overlapped by the lateral margin of the frontal in an interdigitating suture, although the degree of overlap may be exaggerated by deformation of the skull. Posteriorly, the frontals taper to a tip. Originally, the posterior tip of the left frontal probably inserted between the anterior tips of the parietals; however, deformation has pushed both parietals to the right and the frontal overlaps the left parietal. Information from other specimens demonstrates asymmetry in the frontals and variability in their contact with the parietals. The supraorbital lateral line continues from the nasal onto the lateral edge of the frontal as seven pores, the most anterior being an elongate groove.

The **postfrontal** (Figs. 3A and 4) is a crescent-shaped bone forming the dorsal margin of the orbit. In transverse section it is thickest laterally and thins medially. The medial margin of the postfrontal is overlapped by the parietal and frontal; however, the degree of overlap is likely exaggerated in MGUH-VP-8160 because deformation has pushed the skull roof (particularly the parietals) to the right, over the postfrontal, obscuring the posterior half of the element.

The **postorbital** is a tear-drop shaped bone that is broadest posteroventrally and tapers anterodorsally (Figs. 3A and 4). The bone forms the posterior margin of the orbit; transverse CT scans demonstrate the postorbital is mediolaterally thickened around the orbit and thins posteriorly. The anterior tip of the postorbital approaches but does not contact the posterior tip of the postfrontal in MGUH-VP-8160; this contact has been broken by deformation and the original suture type is uncertain. The contacts between the dorsal margin of the postorbital and the lateral margins of the parietal and supratemporal appear to be simple butt joints both in external view and in transverse CT scans. The lateral line from the jugal continues onto the postorbital as an elongate groove instead of individual pores.

In transverse section, the **parietals** (Fig. 4B) are depressed and thin near the midline and thicken at their lateral margins as described by Clack [10] [12]. The skull of MGUH-VP-8160 has been deformed so that the left parietal has been pushed to the right and anteriorly, resulting in substantial overlap of the right parietal by the left parietal. In MGUH-VP-8158, the midline contact between the parietals posterior to the parietal foramen is a simple butt joint (Fig. 3E); this is also the case in external view of the isolated skull in MGUH 29019 while the parietal-parietal contact anterior to the foramen in this specimen appears to be interdigitated [12]. In MGUH-VP-8160, there is a prominent semicircular embayment along the medial edge of the right parietal, representing half of the parietal foramen. The left half of this opening is obscured and has been pushed anteriorly. The parietal tapers anteriorly to a tip that probably inserted between the posterior tips of the postfrontal (laterally) and frontal (medially). Deformation has pushed the right parietal over the right postfrontal in MGUH-VP-8160; thus, the original morphology of these contacts is unclear. The posterolateral edge of the parietal is overlapped by the anteromedial margin of the supratemporal in a strongly interdigitated suture, clearly visible in CT scans of MGUH-VP-8158. We found that the posterior margin of the parietals in MGUH-VP-8158 are extensively overlapped by the anterior margins of the postparietals in an interdigitated suture that is anteriorly convex in external view, resulting in the parietals tapering to posteromedial tips that insert between the postparietals (Fig. 3E). (This morphology differs from that of MGUH-VP-8161 in which the parietal-postparietal contact appears to be transversely straight.)

The arrowhead-shaped **supratemporal** (Figs. 3E and 4B) of *Acanthostega* is unique among tetrapods [2], [10], [12]. The supratemporal tapers posterolaterally forming a pointed process between the squamosal (laterally) and tabular (medially). The anteriorly convex posterior margin of the supratemporal contacts the anterior edge of the tabular along its entire length; CT scans demonstrate that this contact is vertical and highly interdigitated in both MGUH-VP-8158 and MGUH-VP-8160. In MGUH-VP-8158, the medial margin of the supratemporal is overlapped by the anterolateral margin of the postparietal in a short, interdigitated contact (Fig. 3E). Additionally, CT scans of MGUH-VP-8160 reveal a posteriorly-directed tongue of the supratemporal that underlaps the tabular and both buttresses and contributes to the posteriorly-facing facet that clasped a process from the braincase.

The **tabular**, bearing the distinctive spine of *Acanthostega* (Fig. 4B), has been previously described in detail [2], [10], [12]. The free tip of the spine has been broken and laterally displaced in MGUH-VP-8160. Medial to this tip there is a pronounced, U-shaped embayment in the rear of the cranium. The posterior face of the tabular is dominated by a posteriorly facing facet which presumably articulated with the opisthotic [16]. The dorsal and lateral margins of the facet are delimited by a pronounced lip; this lip continues onto the lower part of the facet, which is formed by the supratemporal. The medial margin of the tabular meets the lateral margin of the postparietal in a vertical, strongly interdigitated suture in MGUH-VP-8158.

Among the specimens scanned in this study, only the left **postparietal** of MGUH-VP-8158 is complete, undeformed and preserves all surrounding contacts (Fig. 3E). The postparietals of MGUH-VP-8158 have straight posterior, lateral and medial margins, and an anteriorly convex anterior margin, and form the posterior margin of the cranium. The postparietal is strongly joined to all adjacent bones, including its counterpart across the midline, by interdigitating contacts. The central depression of the parietal continues onto the postparietals, which feature raised lateral margins.

### Palate

The right palate (along with portions of the left premaxilla, vomer and pterygoid) is completely preserved in MGUH-VP-8160 and all information in this section is derived from this specimen. The palate of *Acanthostega* is primarily composed of the pterygoid and ventral braincase; the vomer, palatine and ectopterygoid form the narrow lateral margins of the palate [10]. The palate is broad and largely closed; openings include the midline interpterygoid vacuity (accommodating the parasphenoid), anterior palatal vacuity (enclosed by the premaxilla and vomer), choana, and the subtemporal fossa (Fig. 4C).

In ventral view, the **vomer** is widest at the level of the large vomerine fang and its replacement pit (Fig. 4C). In transverse section, the vomer is L-shaped, with a ventrally projecting lamina (referred to here as the “lateral ridge”, supporting the palatal teeth) making up one leg of the L; the other leg is formed by a tapering, medial lamina that overlies the pterygoid. A short, rounded lateral shelf articulates with the medial shelves of the premaxilla and maxilla.

The anteromedial process of the vomer is better preserved on the left side of MGUH-VP-8160, overlapping an internal shelf of the premaxilla in an interdigitating suture. This anteromedial process forms the medial margin of the anterior palatal fossa, which accommodated the large fangs of the lower jaw. The anterior processes of the pterygoid underlie the anteromedial process of the vomers. The first four vomerine teeth are small and borne on the lateral ridge. The tooth row is then interrupted by a single, large fang; the body of the vomer (seen in CT cross-section) thickens to support this large tooth. Posterior to the fang is a deep replacement pit bounded laterally by the ridge. The lateral ridge posterior to the replacement pit bears at least seven more small teeth. (Although round in cross section and medially curved, the vomerine and palatine teeth are not posteriorly recurved, unlike the marginal dentition.) Posterior to the replacement pit, the lateral margin of the vomer is embayed, forming the internal wall of the choana (Fig. 4C). The short lateral shelf of the vomer articulates with the medial shelf of the premaxilla. The suture between the vomer and palatine is dorsoventrally and mediolaterally expanded. In ventral view, it originates near the posterior margin of the choana and arches medially and anteriorly. The vomer tapers posterolaterally to a point that contacts the maxilla (ventrally) and lacrimal (anterolaterally) in smooth butt joints.

In ventral view, the **palatine** is a tear-drop shaped bone, being mediolaterally wide anteriorly and tapering posterolaterally (Fig. 4C). The anterior margin of the palatine is anteriorly convex and fits into the posterior surface of the vomer, forming a strong joint. The lateral ridge of the vomer continues onto the palatine, bearing four small palatal teeth before the toothrow is interrupted by a large fang and replacement pit. Posterior to this replacement pit, the lateral ridge bears at least ten more palatal teeth. As in the vomer, the medial lamina of the palatine overlaps the pterygoid along its entire length. A short, rounded lateral process of the palatine articulates dorsal to the medial shelf of the maxilla. The posterior process of the palatine also contacts the internal surface of the jugal but this may be due to deformation. We found that the posterior margin of the palatine ventrally laps the anterior margin of the ectopteryoid in a narrow scarf joint directed anteromedially in ventral view.

The **ectopterygoid** is a long, mediolaterally narrow bone (Fig. 4C). The ventrally-projecting lateral ridge of the palatine continues onto the ectopterygoid, gradually decreasing in height posteriorly. The lateral ridge bears ten teeth, which increase in size posteriorly, followed by a gap in the tooth row; it is unclear if this gap is original or due to breakage. There are an additional 14 teeth posterior to this gap, which decrease in size posteriorly. Unlike the anterior palatal teeth, which are medially curved, the ectopterygoid teeth are vertically oriented. The thin medial lamina of the ectopterygoid overlaps the pterygoid along most of its length; posteriorly, the lateral edge of the pterygoid separates from the ectopterygoid to form the lateral margin of the subtemporal fossa (Fig. 4C). The contact between the lateral surface of the ectopterygoid and medial surface of the maxilla is a nearly vertical butt joint. The dorsal surface of the ectopterygoid contacts the ventral edge of the jugal in a butt joint.

The **pterygoid** is the largest bone of the palate and composed of anterior, vertical and quadrate processes (Figs. 3B and 4C). In transverse section, the anterior pterygoid is dorsoventrally thin with a thickened medial margin; this thickening becomes more pronounced posteriorly. The anterior pterygoid is dorsally arched in transverse section; at the level of the interpterygoid vacuity (Fig. 4C) it becomes ventrally arched. The anterior tips of the pterygoid underlie the vomers at the midline and its thin lateral margin underlaps the medial edges of the vomer, palatine and ectopterygoid. The pterygoids contact each other at the midline in a simple, vertical butt joint. Posteriorly, there is a gap in the midline between the pterygoids to accommodate the parasphenoid. The ventral surface of the anterior pterygoid is denticulated except at the midline; the dorsal surface is smooth.

The middle of the pterygoid is dominated by the basal articulation and endochondral component of the palatoquadrate (epipterygoid). This component is fused to the body of the pterygoid; no suture can be seen in CT scans. The medial margin of the pterygoid becomes thick and rounded in cross-section approaching the basal articulation, eventually forming the socket with which the basipterygoid process articulates. This socket is divided into two faces, as suggested by Clack [13]: a kidney-shaped posteriorly facing surface separated by a distinct groove from a medially facing, semicircular surface. The external margins of both faces are delimited by a prominent, raised lip of bone. The lateral margin of this bony lip is continuous with a longitudinal ridge that continues onto the quadrate ramus of the pterygoid (Fig. 3B). This ridge, along with a pronounced groove medial to it, marks the boundary between the body of the pterygoid and the epipterygoid. A sheet of bone (= ascending ramus of Jarvik [2]) extends medially and dorsally from the basal articulation towards the skull roof (Fig. 3B). Anteriorly, this sheet is reinforced by a thickened column of bone, the columella cranii [2]; posteriorly it becomes a thin lamina. This vertical sheet continues posteriorly nearly to the end of the pterygoid, terminating as a thickened flange contacting the internal surface of the squamosal in a rounded butt joint.

The quadrate ramus of the pterygoid is dorsoventrally thickened compared to the anterior half, and its lateral margin, forming the medial margin of the subtemporal fossa, is thickened and turned dorsally. The quadrate ramus of the pterygoid terminates in a rounded posterior margin that is applied to the medial aspect of the quadrate. There is a slight depression on the ventral surface of the quadrate ramus bounded by rounded medial and lateral ridges (Fig. 4C), possibly marking a muscle attachment site; the dorsal surface is dorsally concave.

Scan resolution was high enough to capture denticles on the ventral surface of the pterygoid; however, shagreen lateral to the vomerine/palatine/ectopterygoid teeth or on the ventral surface of the posterior pterygoid [13] could not be resolved.

### Braincase

Little of the braincase is preserved in MGUH-VP-8160. Only the parasphenoid, basisphenoid, basioccipital, and left stapes are described here. Some bone fragments are present in the matrix between the anterior palate and right facial bones; these may represent either the sphenethmoid or sclerotic plates and reveal no anatomical information. There is no sign of an ossified nasal capsule. Segmentation of the dorsal surface of the basioccipital was difficult as a large amount of high density precipitates are present in this area, obscuring fine details in CT scans; it is possible that fragments of the otic capsule and exoccipital are present in this area but cannot be resolved in CT scans.

The **parasphenoid** (Fig. 4C) is well-preserved in MGUH-VP-8160 with the exception of a transverse break and some displacement near its posterior end. The parasphenoid is widest at its rounded, posterior end, tapering to a point anteriorly. Scans show that the cross-sectional geometry of the anterior parasphenoid is that of an I-beam; both ventral and dorsal surfaces bear raised lateral margins bounding a midline depression. Posteriorly, the dorsal depression disappears and only the ventral surface features a midline depression. The anterior parasphenoid occupies the midline gap between the pterygoids but does not contact them. CT scans reveal a curved, smooth suture between the dorsal surface of the posterior parasphenoid and the ventral surface of the basisphenoid, the first time this contact has been described.

Although displaced, it appears that the majority of the **basisphenoid** is present in MGUH-VP-8160 (Figs. 3B and 4C), although the left basipterygoid process is damaged. It is a transversely expanded rectangle in ventral view. The anteriorly-projecting basipterygoid processes are widest at the basal articulation and taper posterolaterally. We found that the articular surface of the right basipterygoid process is concave and bifaceted—a larger anteriorly-directed face and a smaller laterally-directed face—matching the facets of the basal articulation of the pterygoid. Well-defined grooves, marking the course of the internal carotid arteries, separate the basipterygoid processes from the rest of the basisphenoid. A shallow concavity between the basipterygoid processes articulates with the parasphenoid. The lateral wings of the basisphenoid overlying the basioccipital [13], [16] are not present in MGUH-VP-8160. CT scans reveal that the dorsal surface of the basisphenoid bears a pair of circular depressions. Other details of the dorsal or lateral surfaces of the basisphenoid are not clear. Opening of the ventral cranial fissure and displacement of the basisphenoid and basioccipital reveals an anteriorly-concave, oval-shaped facet surrounded by raised lip on the posterior surface of the basisphenoid.

Only the ventral surface of the **basioccipital** (Fig. 3B) is described as segmentation of the dorsal surface proved impossible due to the presence of high density precipitates. The basioccipital is longer than it is wide and the ventral surface features an elongate central concavity bounded laterally by ridges; these ridges are low anteriorly, increasing in height posteriorly. The anterior face of the basioccipital is rounded and anteriorly convex, and would have articulated with the corresponding facet on the posterior face of the basisphenoid.

The **stapes** of *Acanthostega* was first described by Clack [14] and represents one of the earliest known tetrapod stapes. The distal end of the stapes was presumably cartilage-finished [14]; the ossified portion is short and robust. The distal portion of the left stapes is exposed in MGUH-VP-8160; segmentation of CT data revealed the medial head (stapedial footplate) of the stapes, which originally articulated with the fenestra vestibuli. The single head of the stapedial footplate is the thickest part of the stapes, which is constricted in its center and expanded at the distal end, thus being hourglass-shaped in anterior or posterior view. In medial view, two subcircular facets are visible on the stapedial head of MGUH-VP-8160, as described by Clack [15] for CAMZM (UMZC) T1300a. In anterior view, a ridge crosses the stapes obliquely, beginning ventrally at the stapedial footplate and terminating near the dorsal margin distally, resembling illustrations of the right stapes by Clack [11], [14]. The posterior surface of the stapes is flattened. The stapedial foramen cannot be visualized in CT scans. The preserved distal head of the stapes features a kidney-shaped, strongly concave facet surrounded by a raised lip of bone with an overhanging dorsal margin.

### Lower jaw

The lower jaw of *Acanthostega* consists of a core of Meckelian cartilage and bone surrounded by external dermal bones. The lateral face is made up of the dentary and four infradentaries (splenial, postsplenial, angular and surangular); the medial face is made up of the prearticular, three coronoid bones and the adsymphysial. The complete right lower jaw of MGUH-VP-8160 measures 109 mm in length. In lateral view, the ventral margin of the lower jaw is smoothly curved; the dorsal margin follows a similar curve anteriorly but straightens posteriorly, resulting in the anterior third of the jaw being ‘hooked’ (Fig. 5A). There is no surangular crest, although the posterior end of the posterior coronoid is elevated above surrounding bones. The lower jaw is dorsoventrally tallest at the level of the angular-surangular contact and tapers anteriorly, expanding slightly at the symphysis. In medial view (Fig. 5B), the Meckelian fenestra opens as a slot in the center third of the lower jaw between the prearticular (dorsally) and the splenial and postsplenial (ventrally). The Meckelian fenestra persists as a very narrow gap between the prearticular and the angular and surangular in the posterior third of the jaw. In dorsal view, the lateral and medial margins of the lower jaw are parallel along its entire length; the mandibular adductor fossa occupies the posterior third of the lower jaw (Fig. 5D). Deformation of the skull of MGUH-VP-8160 has shifted the left dentary anterior to its counterpart. In contrast, the lower jaws of MGUH-VP-8158 are incomplete but minimally deformed, preserving the symphysis in articulation. Thus, most of the information below is derived from MGUH-VP-8160, with detailed information from the symphysial region drawn from MGUH-VP-8158 (see section titled “Mandibular symphysis of *Acanthostega”* in Discussion).

The **dentary** is long and narrow. In lateral view (Figs. 3A and 5A), it is tallest anteriorly, tapering to a point posteriorly; the dentary does not contribute to the margins of the mandibular adductor fossa. As described by Ahlberg and Clack [17], the lateral aspect of the dentary features a longitudinal ridge that divides the external surface into two faces. In dorsal view, the lateral and medial margins are parallel for much of its length, tapering posterior. There is a notable medial expansion behind the symphysis (which bears large fangs) followed by an embayment that separates this expansion from the anterior tip of the dentary. In medial view, the dorsal margin of the dentary features a strong medial shelf (buttressing the marginal teeth) along most of its length. The anterior third of the ventral margin is inturned; together with the medial shelf, it forms a canal within the dentary that housed the Meckelian cartilage. Both the dorsal and ventral margins thicken as they approach the symphysis before terminating behind the embayment in the dentary. In transverse section, the anterior dentary is V-shaped (the apex pointing dorsolaterally) while the central portion is shaped like an inverted L. There are 58 marginal teeth preserved in the right dentary of MGUH-VP-8160; however, there are anterior gaps that, in the left dentary, are occupied by teeth, suggesting an original tooth count of over 60. The marginal dentary teeth are generally uniform in size, decreasing slightly in height posteriorly and anteriorly; however, the anterior three to four teeth are larger. The medial expansion of MGUH-VP-8160 bears a single large fang medial to marginal teeth four and five; in MGUH-VP-8158, two large fangs are present in the left dentary, with a single fang on the right.

The anterodorsal tip of the dentary diverges from its opposite at the symphysis; ventral and posterior to this tip, there is a dorsoventrally and anteroposteriorly short, flat contact between the dentaries. Scans show that the medial shelf of the anterior dentary meets the adsymphysial in an interdigitated suture. Posteriorly, the shelf thickens and becomes rounded, joining the anterior and middle coronoids in a tongue and groove joint. The medial shelf of the dentary overlaps the lateral lamina of the anterior half of the posterior coronoid; posteriorly, the contact between the dentary and posterior coronoid becomes a simple butt joint. The ventral margin of the dentary contacts the dorsal edge of the lateral lamina of the splenial. Anteriorly, the contact is strongly interdigitated but posteriorly the contact becomes a simple butt joint. The ventral margin of the dentary laterally overlaps the anterior process of the postsplenial, which is hidden in lateral view by the splenial. Once the postsplenial becomes visible in lateral view, its contact with the dentary becomes a butt joint. Posteriorly, the ventral margin of the dentary overlaps the dorsal margins of the angular and surangular, before tapering to a point that laterally overlaps the surangular.

The **splenial** is an elongate bone forming the anteroventral lower jaw in lateral and medial views (Figs. 3B and 5). In transverse section, it is Y-shaped anteriorly and V-shaped posteriorly. One lamina is dorsally-directed (= mesial lamina of Ahlberg and Clack [17]) and the other lamina is laterally-directed; the sharp apex points ventromedially. Anteriorly, there is a pronounced ventral flange (which gives this section of the splenial a Y-shape in transverse section), the medial surface of which is deeply concave. The anterior end of the dorsal lamina of the splenial bifurcates into dorsal and ventral processes. The dorsal process thickens onto a buttress that contacts the adsymphysial in an interdigitating suture. The ventral process bears a rounded, medially-facing pit, interpreted as the attachment site for a ligament [17]. CT scans reveal that ventral to this pit, the medial surface of the splenial of MGUH-VP-8158 meets its counterpart at the symphysis in an interdigitated suture that is anteroposteriorly long. The lateral surface of the ventral process of the splenial is applied to the medial face of the dentary. Posterior to the symphysis, the vertical lamina of the splenial thins and medially overlaps the anterior process of the prearticular. The vertical lamina bifurcates posteriorly into dorsal and ventral processes. The dorsal process is short, overlaps the prearticular, and terminates in a rounded tip. The ventral process is longer, terminating as a pointed tip that ventrally laps the postsplenial, with faint interdigitations posteriorly. The notch between the dorsal and ventral processes is rounded and forms the anterior margin of the Meckelian fenestra.

Scans show that the lateral lamina of the splenial ventrally laps the postsplenial, hiding an anterior process of the postsplenial from lateral view. At least 14 pores of the mandibular lateral line canal are visible along the ventral margin of the splenial, continuing posteriorly onto the postsplenial.

The second in a series of infradentaries, the **postsplenial** is elongate and tallest at its midsection (Figs. 3A, 3B and 5). The bone is gently ventrally arched in transverse section. The anterior third of the postsplenial is not visible in lateral view as it is overlapped ventrally by the splenial and dorsally by the dentary. The contact between the postsplenial and angular is complex—anteriorly, the dorsal margin of the postsplenial laterally overlaps the angular, forming an oblique suture oriented anterodorsally in lateral view. As the angular increases in height posteriorly, the postsplenial is pushed onto the ventral and then onto the medial aspect of the lower jaw (Fig. 5C). The postsplenial-angular suture becomes undulating and terminates as a curved butt joint. The ventral margin of the postsplenial forms a sharp edge delimiting the ventral margin of the Meckelian fenestra; the ventral margin of the prearticular approaches but does not contact the postsplenial. Approximately 16 mandibular lateral line pores line the ventral surface of the postsplenial.

In lateral view, the **angular** is an elliptical bone with a curved ventral margin (Figs. 3A and 5A); CT scans reveal a long, tapering anterior process that reaches the level of the anterior coronoid and is extensively overlapped by the dentary (dorsally) and postsplenial (ventrally). In transverse section, the angular is externally bowed along its entire length. The posterodorsal margin of the angular overlaps the ventral margin of the surangular in a curved suture directed anterodorsally. The lateral laminae of the middle and posterior coronoids touch the medial surface of the angular in places, although this appears to be a loose contact. The thickened, ventral margin of the angular forms the ventral margin of the Meckelian fenestra; the prearticular approaches but does not contact the angular. There are approximately 13 mandibular lateral line pores along the ventral surface of the angular.

The **surangular** makes up the posterolateral lower jaw (Figs. 3A and 5A). It features a straight dorsal margin and strongly curved ventral margin. In transverse section, the surangular is gently laterally bowed; posteriorly, the bone thickens to form the retroarticular process. The lateral face of the surangular is ornamented and features a longitudinal ridge on its posterior third that divides the external surface into a small, concave face directed dorsolaterally and a larger ventral face. The posterior tip of the dentary laterally overlaps the surangular and is continuous with this ridge. The surangular has a tapering anterior process that inserts between the dentary (dorsally) and angular (ventrally). There is also a narrow, butt contact between the dorsal margin of the surangular and the ventral margin of the posterior coronoid. The posterior half of the dorsal margin of the surangular forms the dorsal margin of the lower jaw and laterally delimits the mandibular adductor fossa (Fig. 5D). The ventral margin of the surangular is sharp and inturned, forming the ventral margin of the Meckelian fenestra. The prearticular approaches but does not appear to contact the surangular. Posteriorly, the surangular tapers upwards and curves medially, cupping the lateral and posterior surfaces of the articular. A strong ridge along the posterior margin of the surangular continues onto the angular. The dorsal surface of the posterior surangular is strongly concave with a pronounced lateral lip (Fig. 5D). The quadrate does not contact these structures in MGUH-VP-8160, and it is possible this concavity was the site of attachment for a muscle or ligament.

There are 11–12 mandibular lateral line pores on the ventral surface of the surangular that grow larger approaching the posterior tip of the bone and are continuous with a shallow groove on the posterior edge that served as an attachment for a ligament to the suspensorium in life [17].

The **adsymphysial** (Figs. 3B, 3C and 5B) is an elongate bone consisting of a tapering posterior process and a thickened anterior boss. In transverse section, the posterior process is mediolaterally compressed, increasing in height and width anteriorly. We found that the posterior process of the adsymphysial is overlapped dorsally by the anterior coronoid. The posterior process of the left adsymphysial of MGUH-VP-8158 and right adsymphysial of MGUH-VP-8160 bear five teeth, continuous with the tooth row from the anterior coronoid. The medial adsymphysial foramen lies medial to these teeth in MGUH-VP-8158 (it cannot be visualized in MGUH-VP-8160). Anteriorly, the adsymphysial curves dorsally, forming a thickened boss that bears two large fangs and a smaller, anterior tooth. CT scans show that the medial aspect of the thickened boss meets its counterpart across the symphysis in a strongly interdigitated suture. Additionally, the adsymphysials of MGUH-VP-8158 are asymmetric, with the right element featuring a tongue of bone that extends across the midline and posteriorly laps the left adsymphysial. A short tab extends anteroventrally from the boss to overlap a portion of the splenial; this tab is covered in shagreen.

The right **prearticular** forms most of the medial aspect of the lower jaw (Figs. 3B and 5B), being dorsoventrally tallest at the level of the adductor fossa and tapering anteriorly. In medial view, the dorsal margin is gently curved and the ventral margin is more strongly curved. In transverse section, the prearticular is a mediolaterally thin bone; the dorsal margin is thickened and turned laterally and the bone tapers ventrally. A longitudinal band of shagreen covers the dorsomedial face of the anterior prearticular, fading at the level of the posterior coronoid. In external view, this shagreen is strongly delimited from the ventral part of the prearticular; however, no suture is visible between the dorsal and ventral parts of the prearticular in CT scans. CT scans reveal that the prearticular extends anteriorly, internal to the dorsal lamina of the splenial, nearly reaching the anterior tip of the anterior coronoid. The anterior tip of the prearticular closely approaches but does not appear to suture to posterior tip of the adsymphysial. The prearticulars of MGUH-VP-8158 feature a short, anteriorly tapering process that overlies the dorsal edge of the splenial. The laterally turned dorsal edge of the prearticular overlaps the medial shelves of the anterior and middle coronoid, and the anterior half of the posterior coronoid. The contact between posterior half of the posterior coronoid and the prearticular is a smooth butt joint. The posterior third of the dorsal margin of the prearticular forms the medial margin of the mandibular adductor fossa (Fig. 5D). The prearticular overlaps the medial aspect of the articular. The ventral margin of the prearticular approaches but does not appear to contact the surangular. Anteriorly, there is a gap (Meckelian fenestra) between the irregular ventral margin of the prearticular and the angular and postsplenial.

The **anterior coronoid** is anteroposteriorly elongate and mediolaterally narrow in dorsal view (Fig. 5D), tapering anteriorly and posteriorly. In transverse section, the anterior coronoid is shaped like an inverted T. The vertical lamina bears 14 coronoid teeth (plus at least two gaps in the tooth tow), with the largest teeth in the center of the toothrow. Scans of both specimens show that the lateral edge of the anterior coronoid is grooved to receive the medial shelf of the dentary. The posterior margin of the anterior coronoid meets the middle coronoid in a loose joint; in some sections this appears to be a vertical butt joint, in other sections the middle coronoid dorsally overlaps the posterior edge of the anterior coronoid.

The **middle coronoid** is anteroposteriorly elongate and mediolaterally narrow, tapering anteriorly and posteriorly (Fig. 5D); it is slightly longer than the anterior coronoid. Like the anterior coronoid, the middle coronoid is shaped like an inverted T in transverse section, with the medial shelf underlapping the prearticular and a grooved lateral margin receiving the medial shelf of the dentary. The vertical lamina bears approximately 16 teeth, with the largest teeth in positions eight and nine. We found that the posterior margin of the middle coronoid is extensively overlapped by the posterior coronoid.

The **posterior coronoid** is longer than the anterior or middle coronoids (Fig. 5D). The anterior half of the posterior coronoid resembles the anterior and middle coronoids in its cross-sectional geometry and its lateral and medial contacts. Posteriorly, the lateral and medial shelves disappear and the coronoid increases in height; at this level, the posterior coronoid contacts the dentary (laterally) and the prearticular (medially) in vertical butt joints. Additionally, the ventral margin of the posterior coronoid contacts the dorsal margin of the surangular in a simple butt joint. The posterior tip of the posterior coronoid overlies the dorsal margin of the surangular and contributes to the lateral margin of the adductor fossa. The posterior coronoid bears approximately 20 teeth, although several gaps in the toothrow suggest more teeth were originally present. The tallest teeth are in the middle of the row.

CT scans demonstrate that the **articular** (Fig. 5D) of MGUH-VP-8160 is triangular in transverse section (tapering ventrally) and in lateral/medial views (tapering anteroventrally). The articular is extensively overlapped medially by the prearticular and laterally and posteriorly by the surangular. In MGUH-VP-8160, the dorsal margin of the articular protrudes above the dorsal margins of the prearticular and surangular (thus, the top of the articular is visible in lateral and medial views). The posteromedial aspect of the articular is visible between the posterior margins of the prearticular and surangular. In dorsal view, the articular is mediolaterally widest at its midpoint, tapering posteriorly and anteriorly; its anterior tip projects into the adductor fossa along the medial surface of the surangular. Segmentation of CT scans of MGUH-VP-8160 allows the glenoid surface to be described for the first time (Fig. 5D): it is dorsally concave anteroposteriorly, and is delimited anteriorly and posteriorly by prominent raised lips. The anterior joint surface between the quadrate and articular is nearly horizontal. Posteriorly, there are two distinct articulation surfaces: a small horizontal surface and a larger surface directed dorsolaterally. A small lip on the lateral margin of the articular surface fits outside the lateral condyle of the quadrate while the posterior lip contacts the posterior face of the quadrate. These lips would have constrained movement at the jaw joint to simple rotation. The tapering posterodorsal tip of the articular is rugose.

### 3D reconstruction of the *Acanthostega* skull

Early restorations of the *Acanthostega* cranium depict a skull that is U-shaped in dorsal view and flattened in lateral view, with a smoothly rounded snout, flat skull table, and flattened orbits [2]. The quadratojugal projects below the level of the maxillary tooth row and the posterior margin of the skull—formed by the squamosal, preopercular and quadratojugal—is ‘squared off’ in lateral view. Later reconstructions [12] [13] feature more strongly diverging lateral margins of the cranium (resulting in a more V-shaped profile in dorsal view). In lateral view, the snout is tapered and pointed, the orbit is taller, the ventral margin of the quadratojugal is level with the maxillary tooth row, and the posterior margin of the skull is tapered and rounded. A revised reconstruction [19], based on new information from *Ventastega* [18], featured a midline gap between the frontal bones and the nasals and median rostrals, as well as an upturned snout (based on the shape of the lower jaws).

There are notable differences between the 3D digitally reconstructed cranium (Fig. 4) and recent 2D reconstructions [12], [19]. The orbit is relatively smaller in the digital reconstruction: maximum orbital length is 15% of total skull length in the 3D model compared to 19% of total skull length as depicted by Clack [19]. The postorbital region is slightly longer in the digital reconstruction (47% of total skull length) compared to 44% of total skull length as depicted by Clack [19]. In dorsal view, the sides of the cranium diverge more strongly posteriorly, giving the cranium a distinct V-shape. Furthermore, the anterior end of the snout is not smoothly rounded as in all previous reconstructions; instead, there is a distinct ‘break in slope’ anterior to the maxilla-premaxilla contact. In lateral view, the posterior margin of the skull is squared off, resembling the earliest reconstruction by Jarvik [2], although the ventral margin of the quadratojugal is level with the maxillary tooth row as depicted by Clack [19]. Some features of recent reconstructions [19] are supported by the 3D model, such as the upturned snout and the presence of a small, diamond-shaped vacuity between the nasals and median rostrals. Digital manipulation of 3D surfaces revealed no possibility of fitting together the bones of the anterior snout to completely close this gap. Similar vacuities have been reported in *Ventastega* [18] and *Crassigyrinus* [16] and were presumably filled with cartilaginous tissue in life [19].

In contrast, the digital reconstruction of the lower jaw of *Acanthostega* (Fig. 5) closely resembles previous reconstructions [17]: the entire infradentary series is visible in lateral view, while the prearticular dominates the medial aspect of the lower jaw, with little medial exposure of the coronoid bones. The articular is more visible in medial views of the 3D model than in previous reconstructions. Compared to Ahlberg and Clack [17], the anterior half of the lower jaw in the 3D reconstruction is relatively shorter (dorsoventrally) and more strongly curved, producing a distinct ‘hook-shape’. This resembles later reconstructions by Clack [19] and corresponds to the upturned anterior snout of *Acanthostega*.

## Discussion

### Mandibular symphysis and jaw joint of *Acanthostega*

**Fig 6:**
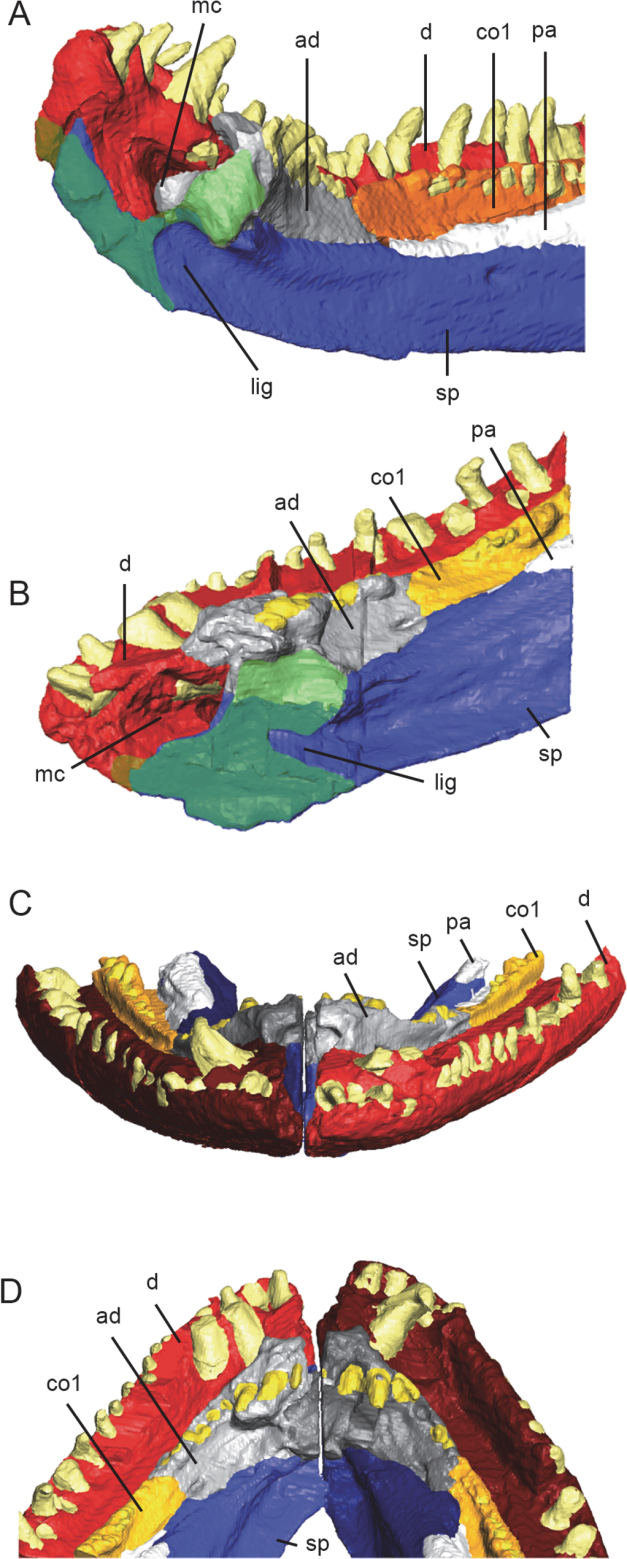
Symphysial morphology of *Acanthostega gunnari*. Surface models of *Acanthostega* illustrating the morphology of the mandibular symphysis. Individual bones are shown in various colours. Medial views of the anterior right lower jaw of MGU-VP-8160 (A) and MGUH-VP-8158 (B), and anterior (C) and posterior (D) views of the articulated anterior lower jaws of MGUH-VP-8158. Green shading in A and B denotes contact surfaces at the symphysis; note the opening of the Meckelian canal enclosed between the adsymphysial, dentary and splenial. Right side bones are shown in darker shades in C and D; note that the anterior portion of the symphysis in MGUH-VP-8158 was sectioned. Anatomical abbreviations: ad, adsymphysial; co1, coronoid 1; d, dentary; lig, ligament pit of splenial; mc, anterior opening of the Meckelian canal; pa, prearticular; sp, splenial.

The mandibular symphysis of *Acanthostega* has been previously figured but much of this area contained broken or sectioned bones (see Fig. 3C in [17]). CT scans reveal new anatomical details of the symphysial region (Fig. 6). As noted in the description, the anterior tips of the dentaries diverge away from the midline. No sutural surfaces are visible in this area of the segmented right dentary of MGUH-VP-8160 (Fig. 6A). Furthermore, the tips of the dentaries do not contact in the articulated lower jaws of MGUH-VP-8158, supporting the presence of a midline gap in the anterodorsal mandibular symphysis (Fig. 6D). Whether this gap was spanned by connective tissue in life is uncertain. CT scans reveal a dorsoventrally and anteroposteriorly short, flat contact between the dentaries posterior and ventral to these tips (Fig. 6A and 6B). This is continuous with an anteroposteriorly long interdigitated suture between the left and right splenials. Anteriorly, the splenial-splenial contact is dorsoventrally short, increasing in height posteriorly. The medially-facing ligament pit of the splenial disrupts this contact; posterior to the ligament pit, the splenial-splenial contact is limited to a dorsoventrally short suture between the ventral flanges of the splenial as figured in cross-section by Ahlberg and Clack [17]. A stronger contact at the mandibular symphysis occurs between the left and right adsymphsials. In anterior view (Fig. 6C), the thickened anterior boss of the adsymphysial arches medially to meet its opposite in a strongly interdigitated midline suture, shown in cross-section by Ahlberg and Clack [17]. A tab of bone from the right adsymphysial of MGUH-VP-8158 extends across the midline to posteriorly overlap the left adsymphysial (Fig. 6D).

The adsymphysial, splenial and dentary join together to enclose the Meckelian canal; this canal opens medially and anteriorly into the mandibular symphysis (Fig. 6A and 6B). Some specimens of *Acanthostega* preserve Meckelian bone near the symphysis [17]; however, no Meckelian bone can be discerned in CT scans of MGUH-VP-8158 or MGUH-VP-8160, suggesting this element was unossified in these specimens. It is unclear whether the Meckelian cartilages were fused across the midline in *Acanthostega*, as occurs in some living lizards [32]. In summary, the mandibular symphysis of *Acanthostega* features strong, bony contacts ventrally and posteriorly, but appears to have been weakly joined and composed primarily of soft tissues anterodorsally.

**Fig 7:**
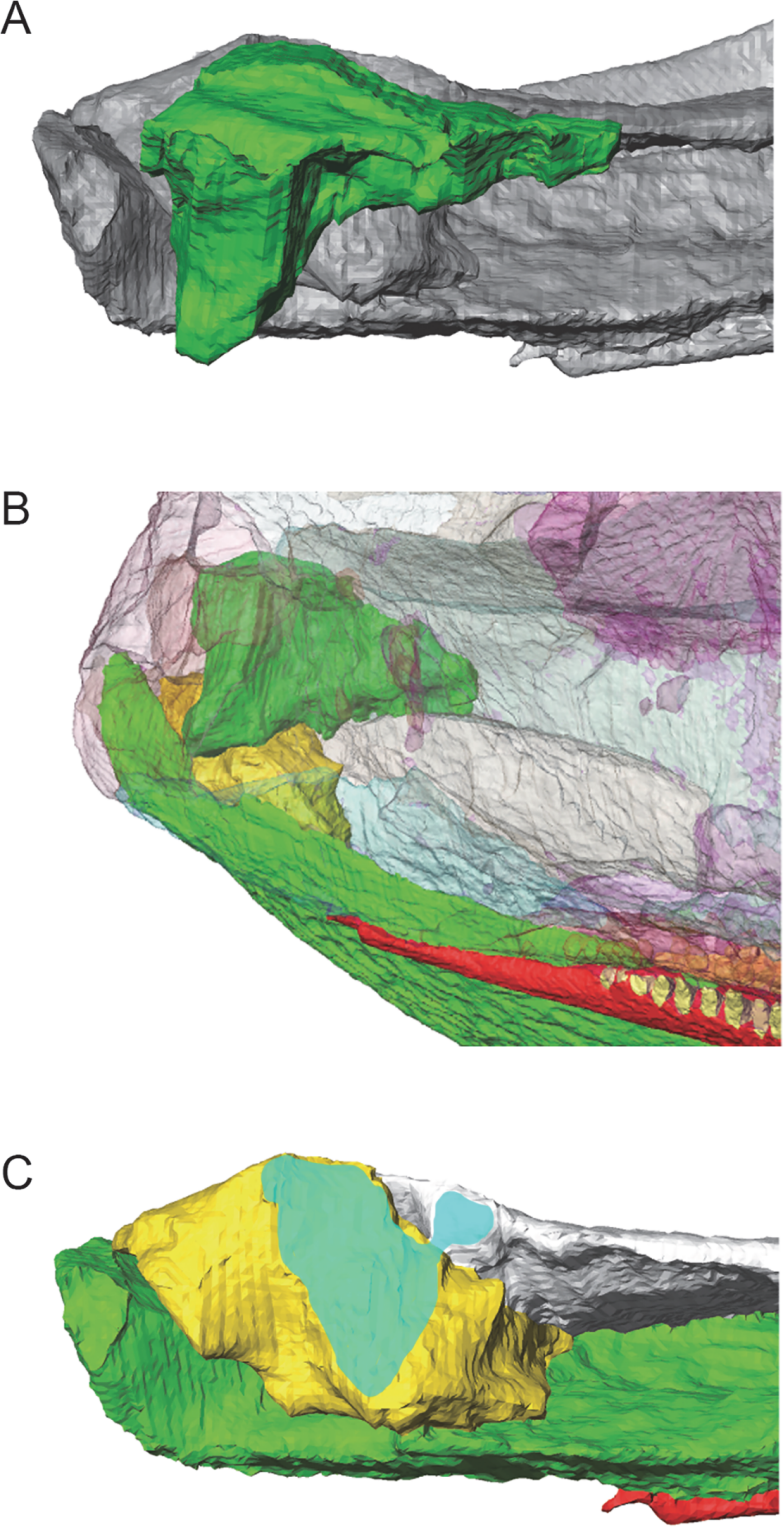
Jaw joint morphology of *Acanthostega gunnari*. Surface models of MGUH-VP-8160 illustrating the morphology of the right jaw joint. The quadrate (green bone) is shown in dorsal view (A) with all surrounding bones of the cranium removed and the lower jaw bones shown in gray. The posteroventral corner of the skull is shown in oblique lateral view (B) with the bones of the cranium transparent to reveal the underlying quadrate (green). The articular (yellow) is show in dorsal view (C) with facets that contact the quadrate shown in turquoise shading.

The jaw joint of *Acanthostega* has never been described as this region is missing, overlain by other bones or tightly articulated in all specimens. CT scans of MGUH-VP-8160 demonstrate that the articular surface of the quadrate is tightly coupled to the prearticular and articular of the lower jaw (Fig. 7). Anteriorly, the groove between the lateral and medial condyles of the quadrate is transversely narrow and deep, and fits onto the dorsal margin of the prearticular. The narrow medial condyle of the quadrate lies medial to the prearticular. The larger lateral condyle of the quadrate contacts the articular of the lower jaw in a joint that is broader (mediolaterally) than it is long (anteroposteriorly). In the center of the joint, the groove in the ventral surface of the quadrate becomes shallow and broad, and the articulation surface is nearly horizontal. Posteriorly, there are two distinct surfaces on the dorsal aspect of the articular: a small, horizontal surface contacts the groove between the condyles of the quadrate and a larger, dorsolateral surface fits against the lateral condyle of the quadrate.

### Cranial and mandibular sutures

Skulls are made up of individual bones joined by collagen fibers at sutures. Sutures assume a number of forms: butt joints that meet at flat edges; scarf joints formed by overlapping bones with beveled margins; and interdigitating sutures, which consist of convoluted, interlocking processes of bone. Additionally, there are a number of less common suture types, such as tongue-and-groove. Because collagen is more elastic than bone, patent sutures decrease overall skull strength [33]; however, *in vivo* [34], [35], [36], [37], [38], [39], [40], *in vitro* [41], [42] and *in silico* [40], [43], [44], [45], [46], [47], [48], [49], [50] studies have suggested that sutures perform a functional role in the skull by modifying or absorbing strain during feeding and other behaviours. As a result, differences in sutural morphology have been linked to particular loading regimes. Butt joints are generally associated with tension or bending while interdigitated sutures are associated with compression [34], [35], [36], [39], [41], with increased interdigitations linked to increased loads [51] and higher strains [35]. Tongue-and-groove joints are associated with resistance to tension [34]. Scarf joints have been associated with torsion [52], [53], [54], [55] and shear [56], with some authors suggesting they represent a morphological compromise to resist both compression and tension [39]. Using correlations between sutural morphology, strain regime and feeding mode in living *Polypterus*, Markey and Marshall [4] predicted terrestrial-style biting (vs. aquatic suction feeding) in *Acanthostega*. In contrast, analyses of functionally relevant mandibular traits demonstrated that *Acanthostega* resembled tetrapodomorph fish [6]. More recently, geometric morphometric and 2D finite element analyses demonstrated that the lower jaw of *Acanthostega* was morphologically and functionally similar to Devonian and Carboniferous aquatic forms [7]. Both studies, focusing on the lower jaw, suggested an aquatic mode of feeding in *Acanthostega*, contradicting predictions based on cranial sutural morphology [4].

**Fig 8:**
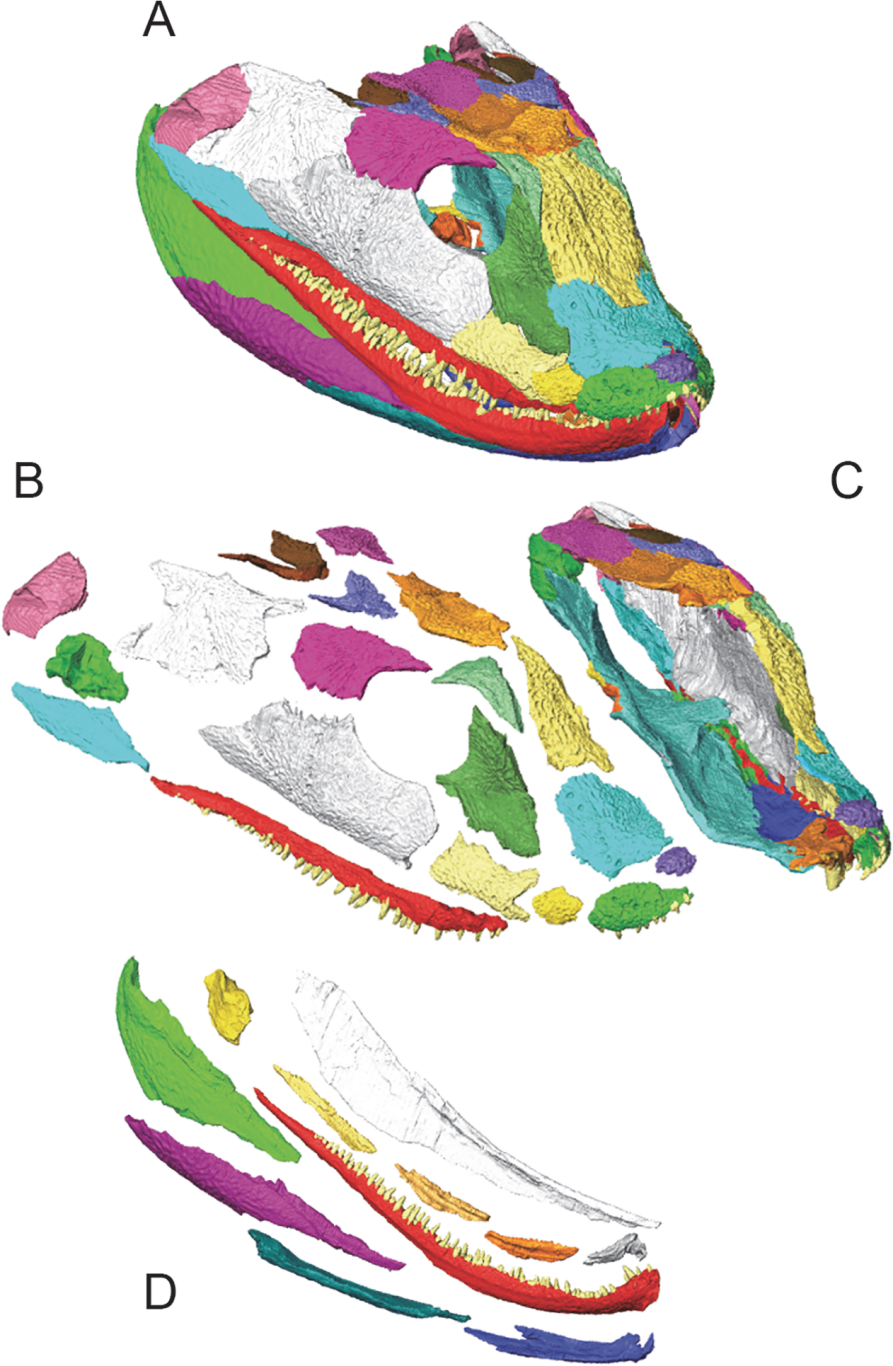
3D reconstructon of the skull of *Acanthostega gunnari*. Articulated cranium and lower jaws shown in oblique right lateral view (A). Right facial skeleton and skull roof shown in “exploded” view to illustrate the nature of sutural contacts (B); the left side of the cranium (braincase omitted) is shown in internal view (C). The right lower jaw in “exploded” view to illustrate sutural morphology. Individual bones shown in various colours.

**Fig 9:**
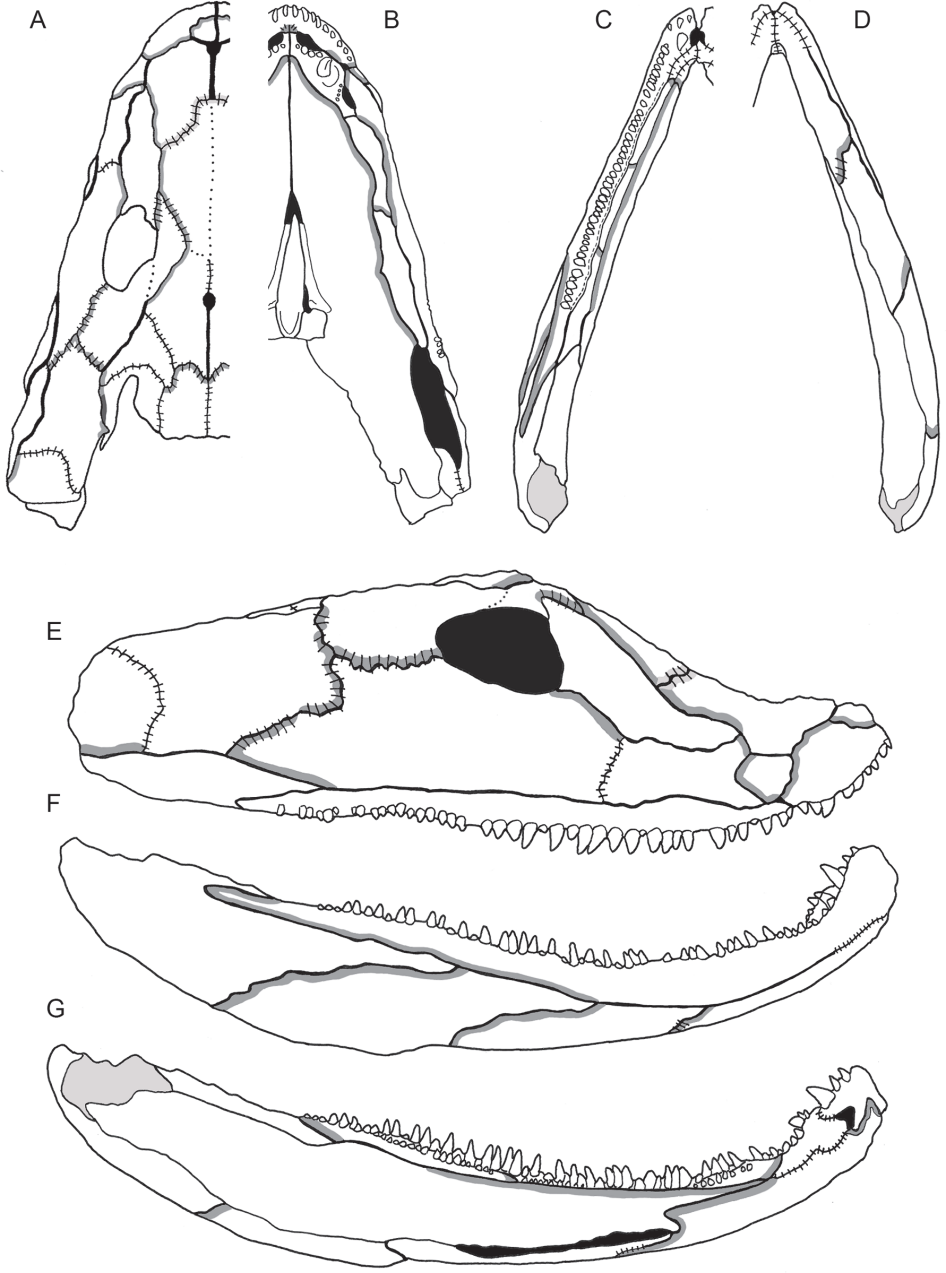
Suture maps of the upper and lower jaws of *Acanthostega gunnari*. Suture maps of the left half of the cranium in dorsal (A) and ventral (B) views, the left half of the lower jaw in dorsal (C) and ventral (D) views, the cranium in lateral view (E), and the lower jaw in lateral (F) and medial (G) views. Solid lines indicate butt joints; medium shading indicates scarf joints and the direction of underlap (but not the extent of underlap); cross hatches indicate interdigitated sutures; dashed lines (C) indicate tongue-and-groove contacts; black shading indicates openings in the skull and jaw. Dotted lines indicate contacts for which sutural morphology is uncertain. The direction of underlap between the nasal and frontal bones is unclear; this is indicated by light shading (A and E). Dark shading in A indicates the unique, curved lapping joint between the tabular and squamosal. The articular is externally overlapped by all surrounding elements and is shown in light shading (C, D, and G).

The detailed morphology of each suture in the skull of *Acanthostega* (as preserved in MGUH-VP-8158 and MGUH-VP-8160) is covered in the description; here we provide an overview of the distribution of different suture types (Figs. 8 and 9) and their possible biomechanical implications. As noted by Clack [12], sutures in *Acanthostega* often exhibit variable morphology along their length and may fit into more than one category—for example, interdigitated scarf joints. The anterior snout of *Acanthostega* features primarily scarf joints, although butt joints are found in the midline (Fig. 9A). Anterior bones generally overlap posterior bones in the snout, consistent with observations made by Kathe [57] in temnospondyls. The posterior facial region and skull table are more firmly sutured than the snout, featuring contacts that are interdigitated, overlapping or both (Fig. 9A and 9E). As noted by Clack [12], the weakest areas of the cranium are the midline of the snout and contacts between the bones of the ventral margin of the skull and those of the cheek region. Nearly all of the palatal bones join in overlapping scarf joints (Fig. 9B). Scarf contacts are also the most common suture type in the lower jaw of *Acanthostega*, with anterior bones overlapping posterior bones (Fig. 9F). Bones at the anterior end of the lower jaw are strongly joined by interdigitated contacts. In contrast, the ventral margin of the dentary meets the postsplenial and posterior portion of the splenial in a weak butt joint. The prearticular sutures rigidly to the coronoid series and the splenial; however, most of its ventral margin is free and does not contact the infradentaries. The distribution of cranial sutures as visualized in CT data was different in several ways from that reported in earlier publications. The following contacts, labeled as butt joints by Clack [12], were found to be scarf joints: median rostral—premaxilla, premaxilla—nasal, anterior tectal—lacrimal, and frontal—prefrontal. The squamosal—preopercular and lacrimal—jugal—contacts are interdigitated. These differences result in the snout and sides of the skull of *Acanthostega* being more strongly sutured than previously reported.

Based on known links between sutural morphology and load regime, we predict compression in the rigidly sutured skull roof and posterior facial skeleton of *Acanthostega*, both of which feature interdigitated sutures. The scarf joints of the anterior snout and cheek region may have acted to resist torsion and shear as forces generated at the tooth row were channeled to the skull roof. The medial lamina of the squamosal, underlapping the tabular and supratemporal bones, would have served to both resist and channel compressive stress from the sidewalls of the skull to the skull roof. The long butt joint between the maxilla and bones of the cheek region may have resisted vertical bite force as well as lateral bending stress produced by struggling prey, as suggested by Clack [12]. However, the suggestion of flexibility in the cheek region [12] seems unlikely given the extensive overlaps and interdigitations between these bones. The scarf and butt joints of the palate would have resisted torsional and tensile stresses, such as those produced during unilateral biting. The nasals and frontals are joined by overlapping and interdigitated contacts, and the frontal shares similar contacts with posterior bones of the skull roof, suggesting longitudinal compression of these bones during anterior bites. In contrast, the internasal and interfrontal contacts are loose butt joints, suggesting mediolaterally oriented tension in this area. These findings are consistent with strain patterns in the skull roof of *Acanthostega* predicted by Markey and Marshall [4] and, in fact, do not match those generated by terrestrial-style biting.

Extensive scarf joints in the lower jaw of *Acanthostega* suggest a complex loading regime including compression, tension, shear and torsion. The weak butt joints between the dentary and the postsplenial and posterior splenial [12], [17] matches the condition between the opposing maxilla and the cheek region, and may likewise suggesting resistance to lateral forces generated by struggling prey. However, the anterior end of the lower jaw (adsymphysial and surrounding bones, anterior dentary and splenial) are strongly sutured, and both the dentary and adsymphysial feature enlarged fangs in this region. Both of these adaptations suggest *Acanthostega* may have used its anterior teeth to initially seize prey; such adaptations are more consistent with a kinetic inertial mode of feeding than a static pressure system [58], [59].

The morphology of the basal articulation between the pterygoid and basipterygoid processes suggests the presence of a synovial joint between the palate and braincase in *Acanthostega* (assuming soft tissues such as hyaline cartilage, synovial fluid and a ligamentous capsule were once present [60]). Previous studies of skull function in both extant and extinct tetrapod skulls have inferred the potential for cranial kinesis from the morphology of the basal articulation [61], [62], [63]. However, synovial basal articulations occur in living animals with akinetic skulls. Holliday and Witmer [60] identified criteria necessary to infer cranial kinesis in extinct taxa, including synovial basal and otic articulations, permissive kinematic linkages and evidence of protractor musculature. Extensive scarf contacts between the pterygoid and the marginal palatal bones (vomers, palatines and ectopterygoids) as well as sutural morphology between the palatal and facial bones in *Acanthostega* suggest intracranial movements were unlikely in this taxon due to a lack of permissive kinematic linkages. Instead the seemingly synovial basal articulation of *Acanthostega* may mark a site of cranial growth during ontogeny or protected the braincase from forces generated in the facial bones and palate during feeding [60]. Given the similar morphology of the basal articulation in taxa leading to amniotes, we suggest that the presence of a synovial basal articulation is a plesiomorphy not only of diapsids [60] but of Tetrapoda, being secondarily lost in temnospondyls, lissamphibians and mammals.

In summary, sutural morphology in the upper and lower jaws of *Acanthostega* supports the use of the enlarged front teeth in strongly biting and seizing prey, with feeding forces being channeled through the nasals and frontals to the skull roof (and through the adsymphysials and dentaries to the anterior lower jaw). Contacts between the maxilla, cheek region and palatal bones (and the dentary and infradentaries) suggest the smaller posterior teeth were used to restrain struggling prey, possibly during ingestion. In general, we find that sutural morphology in the skull of *Acanthostega* is more consistent with an aquatic mode of feeding (i.e., suction feeding), corroborating results by Anderson et al. [6] and Neenan et al. [7]. However, further biomechanical analyses are necessary to support these predictions.

## Conclusions

The results presented here supplement and amend previous descriptions of the *Acanthostega* skull, including new information on sutural morphology, the anatomy of the jaw joint and implications for jaw movements, and a detailed description of the mandibular symphysis. Sutural morphology is used to infer loading regime and possible feeding behavior in this taxon, although these predictions are being subjected to further biomechanical analyses, including finite element modelling, by the authors. Lastly, CT data and visualization software, coupled with novel methodology, were used to create a 3D digital reconstruction of the skull of this iconic fossil taxon, which exhibits both similarities and differences to previous attempts.

## Supporting Information

S1 Matrix Transformation matrices (in Avizo) for individual bones from the original CT data set of the right side of the skull of MGUH-VP-8160 and skull roof of MGUH-VP-8158 to the 3D digitally reconstructed skull. All bones from the lower jaws were exported in a single piece. Reassembled bones from the right side of the skull were mirrored and transformed in a single piece.
(DOCX)

S1 Model 3D PDF of the retrodeformed skull of *Acanthostega gunnari*. Download the PDF file and click once on the skull to activate. Left-click to rotate the model; right-click to zoom in or out; and hold both buttons to pan. Check or uncheck boxes in the tree on the upper left corner of the viewer to display or hide individual parts.
(PDF)
