# Correction: Descriptive Anatomy and Three-Dimensional Reconstruction of the Skull of the Early Tetrapod *Acanthostega gunnari* Jarvik, 1952

**DOI:** 10.1371/journal.pone.0124731

**Published:** 2015-04-10

**Authors:** The PLOS ONE Staff

There are errors in affiliations for authors Emily Rayfield and Jennfier Clack. The affiliations should appear as shown here: Laura B. Porro^1,2,3^, Emily J. Rayfield^2^, Jennifer A. Clack^3^

1 Structure and Motion Laboratory, Royal Veterinary College, University of London, Hatfield, United Kingdom

2 School of Earth Sciences, University of Bristol, Bristol, United Kingdom

3 Department of Zoology, University of Cambridge, Cambridge, United Kingdom
