# Supplementary material for: Descriptive Anatomy and Three-Dimensional Reconstruction of the Skull of the Early Tetrapod Acanthostega gunnari Jarvik, 1952
Source: PLoS One. 2015 Mar 11;10(3):e0118882. doi: 10.1371/journal.pone.0118882 (PMC4356540; doi:10.1371/journal.pone.0118882)
Supplement: S1 Matrix — All bones from the lower jaws were exported in a single piece. Reassembled bones from the right side of the skull were mirrored and transformed in a single piece. (DOCX) [file pone.0118882.s001.docx]

**Supplementary Information 1.** Transformation matrices (in Avizo) for individual bones from the original CT data set of the right side of the skull of MGUH-VP-8160 and skull roof of MGUH-VP-8158 to the 3D digitally reconstructed skull. All bones from the lower jaws were exported in a single piece. Reassembled bones from the right side of the skull were mirrored and transformed in a single piece.

Right facial skeleton

*Right internasal*

0.752611 -0.456182 -0.474829 0 -0.339549 -0.886727 0.313718 0 -0.564161 -0.0748787 -0.822264 0 102.908 574.048 913.733 1

*Right premaxilla and teeth*

0.752611 -0.456182 -0.474829 0 -0.339549 -0.886727 0.313718 0 -0.564161 -0.0748787 -0.822264 0 102.908 574.048 913.733 1

*Right maxilla and teeth*

0.380007 -0.918346 0.110559 0 -0.883508 -0.324977 0.337346 0 -0.273872 -0.225875 -0.934867 0 365.863 362.32 862.104 1

*Right anterior tectal*

0.852343 -0.431116 -0.296036 0 -0.339549 -0.886727 0.313718 0 -0.397755 -0.166877 -0.90219 0 52.8651 593.527 914.626 1

*Right lacrimal*

0.965406 -0.170616 -0.197149 0 -0.0892831 -0.926772 0.364855 0 -0.244964 -0.334633 -0.909952 0 -133.514 642.626 890.843 1

*Right jugal*

0.994466 -0.104447 -0.0107984 0 -0.0981115 -0.96089 0.258958 0 -0.0374236 -0.256466 -0.965827 0 -232.529 609.268 950.486 1

*Right squamosal*

0.930647 -0.359743 -0.0668402 0 -0.332797 -0.908133 0.254043 0 -0.152091 -0.21418 -0.964881 0 -36.1334 563.325 956.82 1

*Right quadratojugal*

0.867627 -0.493775 -0.058267 0 -0.45246 -0.832694 0.319213 0 -0.20614 -0.250595 -0.94589 0 75.419 583.857 881.71 1

*Right preopercular*

0.930647 -0.359743 -0.0668402 0 -0.332796 -0.908133 0.254044 0 -0.152091 -0.214181 -0.964881 0 -35.2257 565.852 955.857 1

*Right quadrate*

0.795211 -0.594354 0.119926 0 -0.598529 -0.737833 0.312037 0 -0.096974 -0.319915 -0.942472 0 15.3617 631.283 808.511 1

Right skull roof

*Right nasal*

0.803779 -0.344531 -0.485 0 -0.223577 -0.930414 0.290415 0 -0.551313 -0.124995 -0.824887 0 49.1137 598.01 929.818 1

*Right prefrontal*

0.905852 -0.417096 -0.0738015 0 -0.344093 -0.82623 0.446032 0 -0.247017 -0.378649 -0.891973 0 -19.7303 653.584 853.268 1

*Right frontal*

0.734767 -0.676638 -0.0476029 0 -0.54753 -0.633062 0.547208 0 -0.4004 -0.376011 -0.835643 0 121.073 638.473 796.462 1

*Right postfrontal*

0.721281 -0.689451 0.0662751 0 -0.606007 -0.581845 0.542409 0 -0.335405 -0.431398 -0.837497 0 106.757 657.806 777.129 1

*Right postorbital*

0.634477 -0.752792 0.175312 0 -0.772034 -0.606234 0.190895 0 -0.0374249 -0.256465 -0.965829 0 -10.8949 579.846 951.392 1

*Right parietal*

0.775914 -0.626388 -0.0746375 0 -0.552994 -0.732352 0.397328 0 -0.303545 -0.267022 -0.914646 0 135.735 587.76 912.892 1

*Right supratemporal*

0.686354 -0.723598 0.07286 0 -0.713577 -0.650707 0.259599 0 -0.140436 -0.230169 -0.962966 0 47.2676 567.531 944.648 1

*Right tabular*

0.682179 -0.72932 0.0520748 0 -0.72158 -0.660007 0.209077 0 -0.118115 -0.180204 -0.976513 0 24.952 523.885 963.143 1

*Postparietal (+supratemporal, parietal and tabular fragments) from MGUH*-VP-8158

-0.31493 0.0554119 0.0121857 0 0.0499134 0.30327 -0.089082 0 -0.0269742 -0.0857701 -0.30711 0 204.016 -10.0666 589.325 1

Right palate

*Right vomer, palatine and ectopterygoid with teeth*

0.380006 -0.918345 0.110559 0 -0.892295 -0.332457 0.30542 0 -0.243725 -0.214714 -0.945779 0 345.663 351.947 867.287 1

*Right pterygoid*

0.356181 -0.933864 0.0319617 0 -0.88488 -0.326114 0.332618 0 -0.300196 -0.146755 -0.942522 0 353.613 321.804 841.968 1

Left side of facial skeleton, skull roof and palate

First transform: -0.97659 -0.139148 -0.164045 0 -0.140644 0.990058 -0.00252132 0 -0.162765 -0.0206096 0.986449 0 128.813 14.3494 7.86798 1

Second transform: 0.998525 0 0.054299 0 0 1 0 0 -0.054299 0 0.998525 0 51.8155 0 1.76059 1

Braincase

*Parasphenoid*

0.536263 -0.819275 0.202993 0 -0.807464 -0.427926 0.406061 0 -0.24581 -0.381666 -0.891016 0 282.702 458.15 712.241 1

*Basisphenoid*

0.498204 -0.865414 -0.0533931 0 -0.369914 -0.26784 0.889621 0 -0.784192 -0.423464 -0.453569 0 516.716 466.153 379.671 1

*Basioccipital*

-0.0640088 -0.982909 -0.172606 0 -0.506379 -0.117051 0.85433 0 -0.859931 0.142089 -0.490233 0 718.013 68.0233 434.087 1

Lower jaw transformations

*Right lower jaw (exported in one piece)*

0.761154 -0.648007 -0.0270263 0 -0.607678 -0.727108 0.319443 0 -0.226651 -0.226722 -0.947225 0 182.736 520.918 850.803 1

*Left lower jaw (exported in one piece)*

First transform: -0.629333 -0.763521 -0.144787 0 0.648926 -0.61882 0.442655 0 0.427575 -0.184622 -0.884925 0 -282.152 483.791 816.967 1

Second transform: 0.999808 0 0.0196004 0 0 1 0 0 -0.0196004 0 0.999808 0 33.1073 2.44775 -10.3209 1
