# Supplementary figures and images for: Descriptive Anatomy and Three-Dimensional Reconstruction of the Skull of the Early Tetrapod Acanthostega gunnari Jarvik, 1952
Source: PLoS One. 2015 Mar 11;10(3):e0118882. doi: 10.1371/journal.pone.0118882 (PMC4356540; doi:10.1371/journal.pone.0118882)

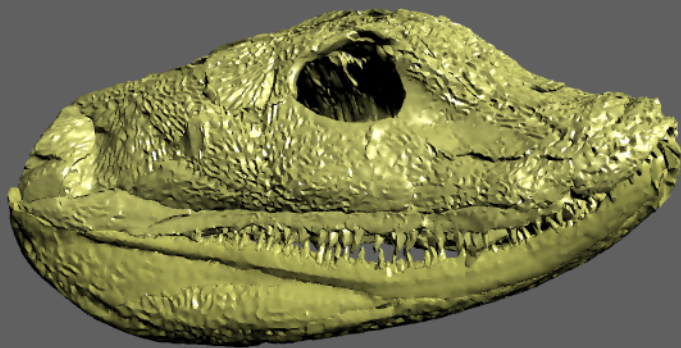

Supplement: S1 Model — Download the PDF file and click once on the skull to activate. Left-click to rotate the model; right-click to zoom in or out; and hold both buttons to pan. Check or uncheck boxes in the tree on the upper left corner of the viewer to display or hide individual parts. (PDF) [file pone.0118882.s002.pdf]
